# N6-methyladenosine (m6A)-connected lncRNAs are linked to survival and immune infiltration in glioma patients

**DOI:** 10.1042/BSR20222100

**Published:** 2023-05-05

**Authors:** Wei Jun Wu, Feng Xiao, Yaping Xiong, Gu Feng Sun, Yun Guo, Xiang Zhou, Guo Wen Hu, Kai Huang, Hua Guo

**Affiliations:** 1 Department of Neurosurgery, The Second Affiliated Hospital of Nanchang University, Nanchang, China; 2 Jiangxi Key Laboratory of Neurological Tumors and Cerebrovascular Diseases, Nanchang, China; 3 Jiangxi Health Commission Key Laboratory of Neurological Medicine, Nanchang, China; 4 Institute of Neuroscience, Nanchang University, Nanchang, China; 5 Department of Neurosurgery, Yichun People’s Hospital, Yichun, Jiangxi, China; 6 Departments of Anesthesiology, The Second Affiliated Hospital of Nanchang University, Nanchang 330006, Jiangxi, China

**Keywords:** Glioma, immune infiltration, LncRNA, M6A regulators, prognosis

## Abstract

**Background:** The connection between m6A-assiociateed lncRNAs and prognosis has been demonstrated in multiple types of tumors. However, potential roles of m6A-assiociateed lncRNAs in glioma is still rare.

**Methods:** We implemented consensus cluster analysis to group the downloaded samples into two subtypes. The least absolute shrinkage and selection operator (LASSO) analysis was used to create a risk model. Additionally, the conjunction between m6A-related lncRNAs and immune cells infiltration was explored by conducting the R package. Ultimately, we inspected the underlying downstream pathways of the two subtypes by performing Gene Set Enrichment Analysis (GSEA). The expression level of m6A-connected lncRNAs in glioma were examined by conducting *in vitro* experiments.

**Results:** We ascertained two subtypes of glioma in line with the consensus clustering of m6A-associated lncRNAs. We confirmed that age, grade, and IDH are related to the two subtypes. Additionally, the immune cells infiltration and immune checkpoint molecules of the two clusters were discussed. A risk signature including AL359643.3, AL445524.1, AL162231.2, AL117332.1, AP001486.2, POLR2J4, AC120036.4, LINC00641, LINC00900, CRNDE, and AL158212.3, was identified using the Cox regression and LASSO analyses. We also verified the prognostic value and discussed the immune cells infiltration and immune checkpoint molecules of the risk signature. *In vitro* experiments verified that the m6A-associated lncRNAs was abnormally expressed in glioma.

**Conclusion:** We elaborated the significant role of m6A-connected lncRNAs in glioma prognosis and immune infiltration and suggest that these key regulators may serve as underlying therapeutic targets to build up the efficacy of glioma immunotherapy.

## Introduction

Glioma is the most prevalent intracranial tumor that has a low overall survival (OS) rate [1]. Although conventional treatments, such as surgery, radiotherapy, and chemotherapy, have achieved great improvements. The prognosis is still poor due to the rapid proliferation and angiogenesis of glioma [2]. Interestingly, many studies have shown that immunotherapy is a promising strategy in treating glioma [3–5]. In consequence, it is imminently needed to excavate underlying molecular mechanism of glioma and explore novel immunotherapeutic targets for glioma.

RNA modifications are reversible and may be closely linked to major biological processes, including stress responses, cell differentiation, and sex determination [6]. In eukaryotes, the m6A modification is the most prevalent RNA modification and there is mounting evidence that highlights the significant function of m6A RNA methylation in RNA expression and metabolism. The activity of m6A RNA methylation has been shown to be tightly interrelated with the malignant progression of tumors [7]. M6A is regulated by methyltransferase complexes and involves three homologous factors, including ‘writers’, ‘readers’, and ‘erasers’ [8]. Interestingly, m6A is installed by the methyltransferases, termed as ‘writers’. ‘Readers’ are regarded as particular RNA binding proteins which can identify m6A and implement biological functions. Demethylase, also called ‘eraser’, removes m6A [9]. Therefore, m6A RNA methylation is a dynamically reversible process. An accumulating body of research has demonstrated that m6A regulators were tightly connected with the tumorigenesis, proliferation, and drug resistance in glioma [10–12].

Long non-coding RNA (lncRNA) is nonprotein-coding RNA, which transcripts length is greater than 200 nucleotides [13]. Interestingly, several studies demonstrated that lncRNA plays significant role in glioma cancer epigenetics, gene expression, and protein translation [14–16]. Importantly, lncRNA can combine with m6A regulators to regulate the occurrence and development of glioma [17,18]. Therefore, it is essential to further our understanding about the role of m6A-associated lncRNA in glioma progression, and particularly, its effect in the regulation of RNA expression and metabolism.

The tumor microenvironment (TME) contains tumor interstitial cells, extracellular matrix, and soluble molecules, and plays a vital part in tumor progression. Several studies have illustrated that immune cells can infiltrate the TME, and therefore, the presence of these cells can be used in the diagnosis, treatment, and prognosis of tumors [19–22]. Interestingly, the composition of TME has been proved to respond to immune-checkpoint inhibitors. Immune-checkpoint inhibitors make use of immune cells’ infiltration in tumor to reactivate effective anti-tumor immunity [23]. In recent years, increasing research has illustrated that immune-checkpoint inhibitors play a crucial part in anti-tumor immunity [24–27].

This research aimed at exploring the correlations between m6A-connected lncRNAs, glioma prognosis, immune-checkpoint molecules and immune cells' infiltration using bioinformatic approaches. We have identified m6A-connected lncRNAs involved in glioma immune microenvironment that can potentially improve glioma immunotherapeutic strategies. Importantly, we implemented the *in vitro* experiments to inspect the aberrant expression of m6A-connected lncRNAs in glioma.

## Methods

**Figure 1:**
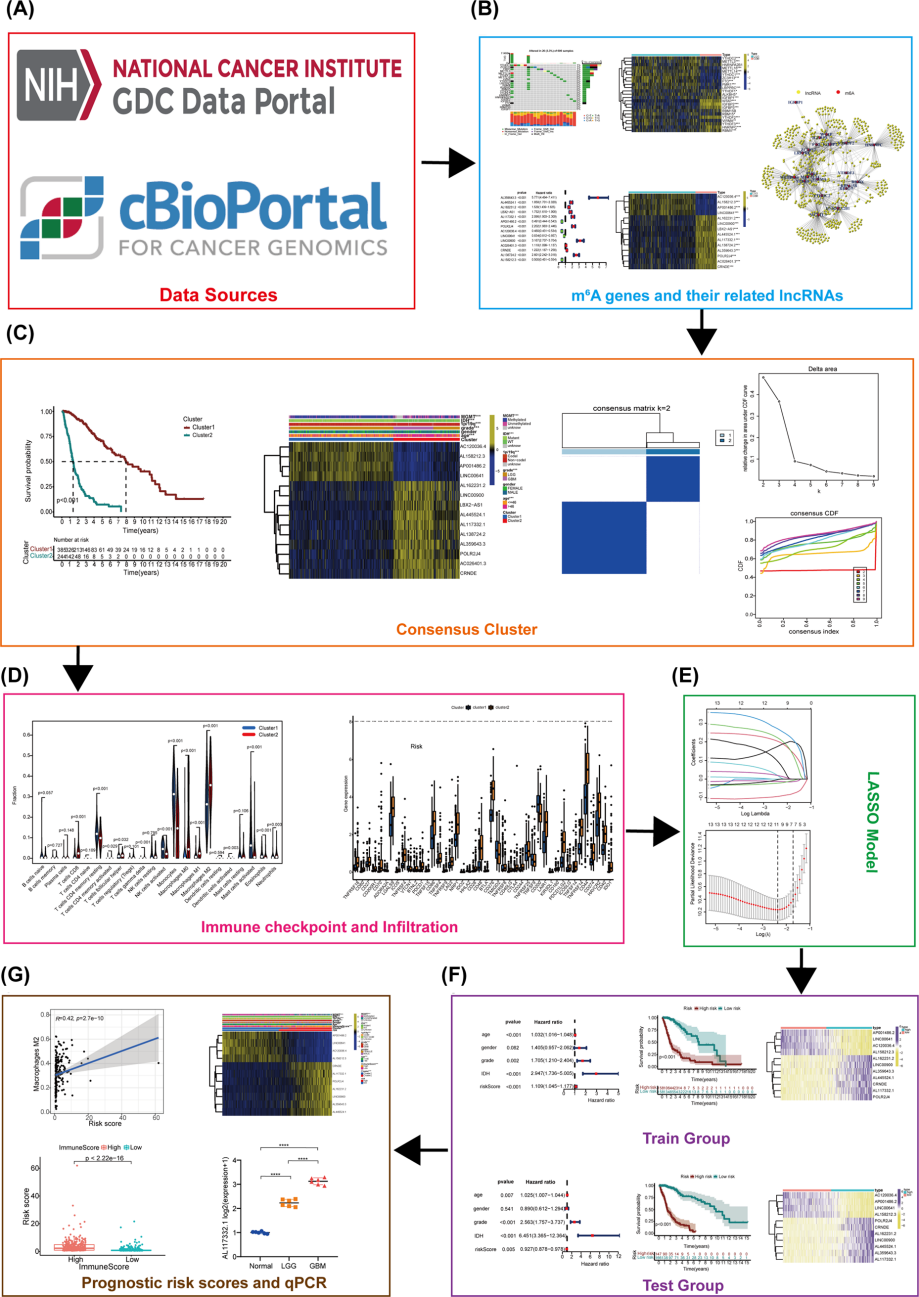
Data analysis flowchart (**A**) Data sources. (**B**) m6A genes and their related lncRNAs. (**C**) Consensus Cluster. (**D**) Immune checkpoint and infiltration. (**E**) LASSO Model. (**F**) Train and Test groups. (**G**) Prognostic risk scores and qPCR.

The data analysis process is displayed in Figure 1.

### Explanation of data collection

We downloaded data from The Cancer Genome Atlas (TCGA) dataset (http://cancergenome.nih.gov/): RNA-seq transcriptome from 698 glioma samples and clinical data from glioma patients. In addition, we supplemented these data with clinical information of glioma patients from cBioPortal dataset (http://cbioportal.org), such as MGMT, IDH, and 1p/19q. The RNA-seq transcriptome data, that were acquired from TCGA dataset, were standardized through fragment per kilobase of exon model per million (FPKM). Subsequently, we matched the clinical information and RNA-seq data of glioma patients. After matching gene expression with clinical data and deleting the OS <30 days, our biometric analysis data included 629 tumor samples (Supplementary Table S1). Additionally, we also included 655 glioma samples in CGGA dataset (http://www.cgga.org.cn/) (Supplementary Table S2). Glioma patients with clinical information were classified into a test subtype and a train subtype in a 1:1 ratio through adopting the ‘caret’ R Package (https://CRAN.R-project.org/package=corrplot). In addition, the somatic mutation data of glioma patients was also obtained from TCGA datasets.

### Exploration of m6A genes and connected lncRNAs

We selected 23 m6A-associated genes (Supplementary Table S3) in line with the former research [28,29]. Next, we acquired the 23 m6A gene expression profiles from the RNA seq transcriptome data. We exercised co-expression analysis by performing the ‘limma’ R package [30] and the filter conditions for screening m6A lncRNA methylation regulators were: ‘corFilter = 0.4’ and ‘pvalueFilter = 0.001’. The expression of co-expression network for lncRNAs was acquired through the ‘igraph’ R package (https://CRAN.R-project.org/package=igraphdata). The signature in 14 m6A-associated lncRNAs was screened by univariate Cox regression. Hazard ratio> 1 manifested an increase in risk, while hazard ratio <1 manifested a smaller risk. In addition, differences in m6A-related genes and their co-expressed lncRNAs between LGG and glioblastoma (GBM) samples were shown in boxplot and heatmap using the ‘limma’ package.

### Analysis of the consensus clustering and prognostic signature

We classified the glioma patients into cluster1 (*n*=385) and cluster2 (*n*=244) using the ‘ConsensusClusterPlus’ package [31]. The OS of the two subtypes was analyzed by conducting the Kaplan-Meier method. The correlation between grouping and clinical factors was shown in a heatmap through the ‘pheatmap’ R package (https://CRAN.R-project.org/package=pheatmap). Additionally, GSEA 4.1.0 was exploited to discover the potential pathway of the two clusters.

We employed LASSO regression analysis to create prognostic risk model of m6A-associated lncRNAs. This is the formula that was executed to measure the risk score [29]:

$$Riskscore=\sum_{i=1}^{n}codfi\times xi$$

The *codfi* is on behalf of the coefficient and *xi* is on behalf of the expression value of every m6A-associated lncRNA. We measured the risk score of every glioma sample in train (*n*=316) and test (*n*=313) cohorts using this formula. The purpose of the test cohorts is to validate the train cohorts. The tumor samples in the train and test subtypes were grouped into low-risk and high-risk subtypes in the light of the median risk score.

### Evaluation of prognostic value of m6A-associated lncRNAs

We assessed the distinct OS between low-risk and high-risk cohorts in train and test groups by conducting Kaplan–Meier analysis method. Afterwards, the receiver operating characteristic (ROC) curve was executed and the area under the curve (AUC) was calculated to analyze the predictive effect of the signature. Risk scores, which were integrated with other clinical features, were evaluated for whether they could act as independent prognostic biomarkers by exploiting Cox regression analyses. Clinical traits in low-risk and high-risk cohorts were displayed in heatmaps by conducting ‘pheatmap’ R package.

### Conjunction between immune cells' infiltration and m6A-associated lncRNAs

We calculated the immune scores of glioma patients by employing ESTIMATE algorithm [32]. To acquire the abundance for 22 types of immune cells in each glioma patient, we implemented CIBERSORT algorithm [33]. We adopted a 1000 permutation algorithm and only analyzed the samples with *P*<0.05. Subsequently, we compared distinct levels of immune cells infiltration between cluster 1 and cluster 2 by clustering and risk scores. We selected 47 immune-checkpoint genes (ICPGs) (Supplementary Table S4) in line with previous studies [34–36]. Additionally, we also compared distinct ICPGs expression between the two clusters. Similarly, we also contrasted different levels of immune cells infiltration and ICPGs expression between low-risk and high-risk subtypes in train and test subtypes.

### Quantitative Real-Time PCR

Six normal brain tissues, LGG, GBM tissues, which obtained from the Second Affiliated Hospital of Nanchang University, were utilized in this research. We isolated total RNA from brain tissues with the Simply P Total RNA Extraction Kit (Bioflux, Beijing, China) and then reverse-transcribed it into complementary DNA with HiScript III-RT SuperMix (Vazyme, Nanjing, China). Then, relative expression of lncRNAs was normalized to GAPDH, and fold change was inspected by employing the 2^−∆∆CT^ method. The primer sequences were displayed in Supplementary Table S5.

### FISH and Immunofluorescence

We performed RNA-FISH with AL117332.1, AL359643.3, AL445524.1, CRNDE, LINC00641, and AP001486.2 specific probe (Alexa Fluor 488, Biosearch Technologies). The cells were fixed in 4% paraformaldehyde for 15 min and then incubated with specific probe overnight at room temperature. Then, the cells were blocked using 3% BSA. Ultimately, we stained the cells using DAPI and imaged by conducting confocal laser scanning microscopy (Nikon, Japan).

### Statistics

We operated all statistical analyses by R language v4.1.3 and GraphPad Prism 8 (GraphPad Software, Inc.). To compare differences between the two cohorts and among multiple cohorts, we implemented the one-way analysis of variance (ANOVA) and default Wilcoxon test. Additionally, the Kaplan–Meier analysis was utilized to evaluate the distinct OS between subtypes. The subgroups, clinical characteristics, risk scores, and abundance of immune cells infiltration, were executed by the Pearson correlation test. The obtained results were deemed to be significant only when *P*<0.05.

## Results

### Differential expression of m6A genes and their connected lncRNAs in glioma samples

**Figure 2:**
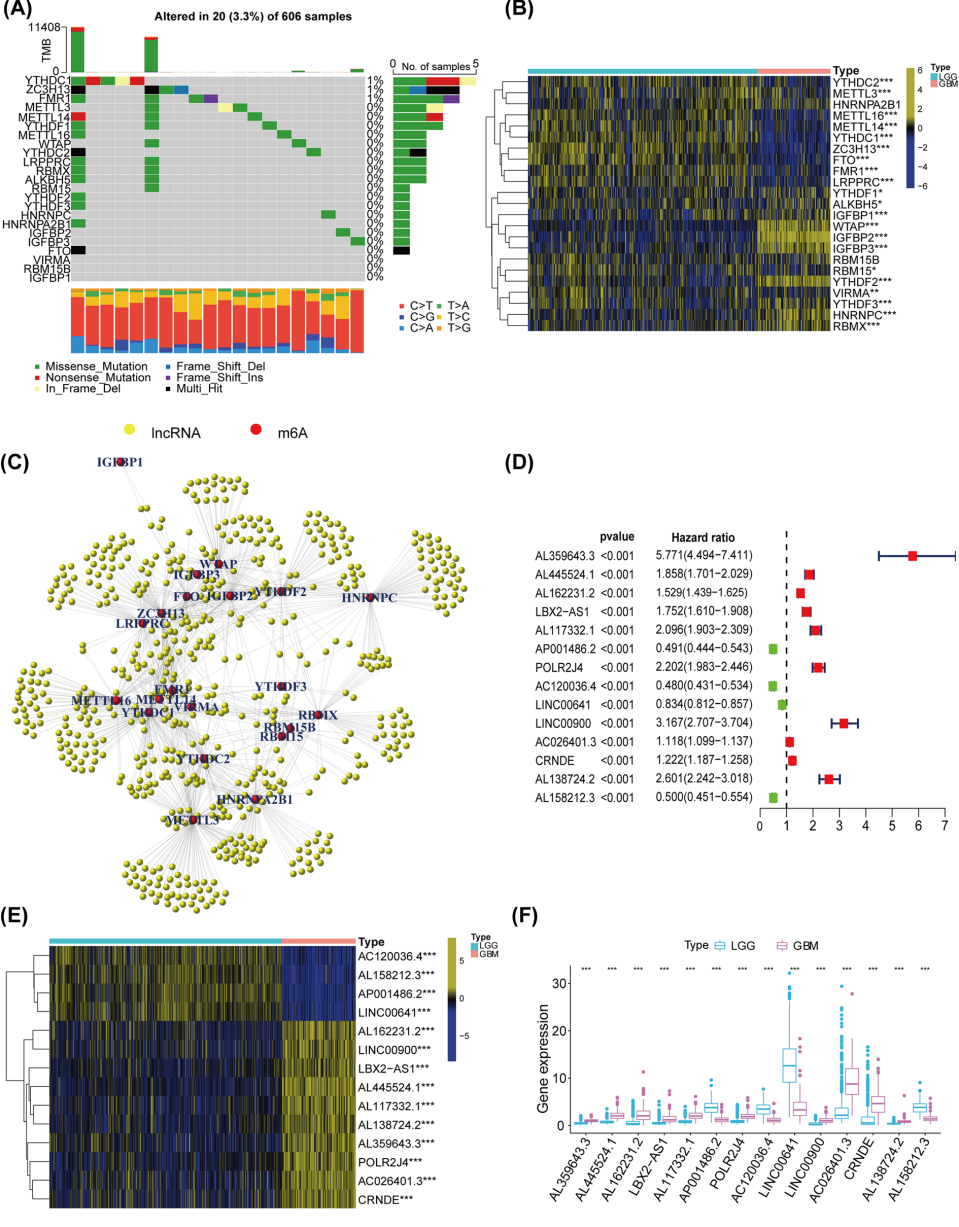
The expression traits and interactions of m6A-associated genes in glioma (**A**) Waterfall blot displayed the mutation of 23 m6A-related genes. (**B**) Heatmap exhibited the overall expression of 14 m6A-conencted genes in LGG and GBM from TCGA datasets. (**C**) The connection between m6A-related genes (red) and the related IncRNAs (yellow). (**D**) Univariate Cox regression analysis for the features of 14 m6A-associated lncRNAs. (**E**) Heatmap showed the overall expression of 14 m6A-connected lncRNAs in LGG and GBM from TCGA datasets. (**F**) Differential expression of the m6A-connected lncRNAs in LGG and GBM was displayed by boxplot. *P*<0.05 (*), *P*<0.01 (**), and *P*<0.001 (***).

We recognized 23 m6A signatures, including 2 erasers, 8 writers, and 13 readers. Firstly, we downloaded the somatic mutations and copy number variations of the 23 m6A regulators in glioma from TCGA. Among 606 matched samples, 20 had mutations in m6A genes with a frequency of 3.3%. The results showed that YTHDC1, ZC3H13, and FMR1 exhibit a similar mutation frequency. However, the remaining 20 m6A regulators did not reveal any mutations in glioma (Figure 2A). In addition, the expressions of the 23 m6A genes in LGG and GBM samples, were also studied by analyzing RNA-seq transcriptome data, that were downloaded from TCGA. The analyzed data included 477 LGG and 152 GBM samples. As shown in the heatmap (Figure 2B), ‘writers’ (WTAP), ‘Readers’ (YTHDF1, YTHDF2, YTHDF3, HNRNPC, IGFBP1, IGFBP2, IGFBP3, RBMX, and ALKBH5) and ‘erasers’ (ALKB5H) in GBM samples were distinctly higher than those in LGG samples. Therefore, m6A regulators may play a crucial part in glioma.

To explore the correlations between the 23 m6A genes and their associated lncRNAs, RNA-seq transcriptome data were analyzed to obtain the 23 m6A regulatory genes that are co-expressed with the lncRNAs. Whereafter, we established a co-expression network to display the connection between m6A signatures and lncRNAs by performing ‘igraph’ R package. The yellow nodes represent lncRNAs, the red nodes are m6A regulators (Figure 2C). The univariate Cox regression analysis was exploited to examine the conjunction between m6A-associated lncRNAs and glioma prognosis. Under the filter condition *P*-value <0.001, we selected the leading 14 m6A-related lncRNAs that were ranked by *P*-value in the univariate Cox regression (Figure 2D). We also studied the differential expressions of the 14 m6A-connected lncRNAs between TCGA LGG and GBM samples. Through their visualization in heatmap (Figure 2E) and boxplot (Figure 2F), we found that most lncRNAs in GBM are higher than those in LGG, except for AP001486.2, AC120036.4, LINC00641, and AL158212.3.

### Consensus clustering of m6A-associated lncRNAs and clinical features in glioma

**Figure 3:**
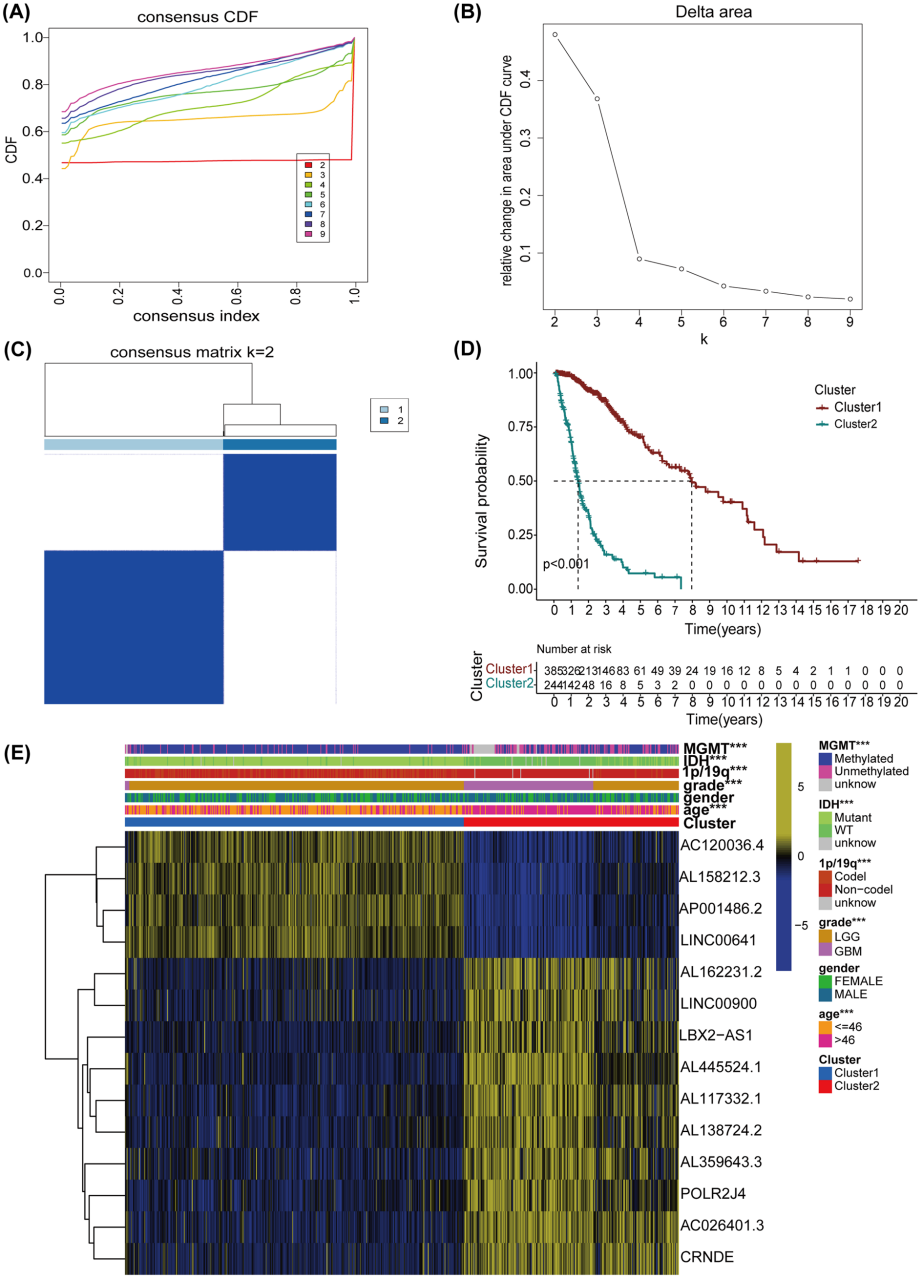
Conjunction between the m6A methylated lncRNAs and prognostic and clinical characteristics of glioma (**A**) Consensus clustering model with CDF for *k* = 2–9 (*k* represents cluster count). (**B**) For *k* = 2–9, relative change in area under the CDF curve. (**C**) Glioma TCGA cohort was categorized as two clusters with *k* = 2. (**D**) The overall survival of glioma patients in cluster 1 and cluster 2 was figured out by Kaplan–Meier analysis. (**E**) Heatmap presented the correlation of cluster1 and cluster 2 with clinical features; *P*<0.001 (***).

We performed consensus clustering analysis through cumulative distribution function (CDF) (Figure 3A,B), with *k* from 2 to 9. We found that *k* = 2 (Figure 3C) was the best clustering parameter compared to others (Supplementary Figure S1) when analyzing the proportion of fuzzy clustering measures and the similarity of m6A-related lncRNAs expression. The 629 glioma patients who were matched by the selected lncRNAs expression and the survival time of patients were segmented into cluster 1 (*n*=385) and cluster 2 (*n*=244). Compared with cluster 1, the OS was lower in cluster 2 (*P*<0.001) (Figure 3D). Interestingly, the expression levels of AL162231.2, LINC00900, LBX2-AS1, AL445524.1, AL117332.1, AL138724.2, AL359643.3, POLR2J4, AC026401.3, and CRNDE in cluster 1 were lower than those in cluster 2. However, the expression levels of AC120036.4, AL158212.3, AP001486.2, and LINC00641 in cluster 2, were lower than those in cluster 1. Subsequently, the clinical features of the two clusters were compared, and we found that MGMT, IDH, 1p/19q, grade, and age were closely interrelated with the cluster analysis (Figure 3E).

Using GSEA, we also displayed potential pathways that were distinct between the two clusters. Under the filter condition normalized enrichment score (NES) >1, Nominal (Nom) *P*-value<0.05, and false discovery rate (FDR) *q*-value<0.25, we found that the main enrichment pathways of cluster 2 were ‘cytokine–cytokine receptor interaction’, ‘nucleotide excision repair’, ‘antigen processing and presentation’, ‘cell cycle’, ‘primary immunodeficiency’, and ‘p53 signaling pathway’ (Supplementary Figure S2A). However, cluster 1 was more related to the main enrichment pathways, including ‘long term depression’, ‘inositol phosphate metabolism’, and ‘WNT signaling pathway’ (Supplementary Figure S2B).

### Consensus clustering of m6A-associated lncRNAs interrelated with differential immune cells' infiltration and immune checkpoint genes expression

**Figure 4:**
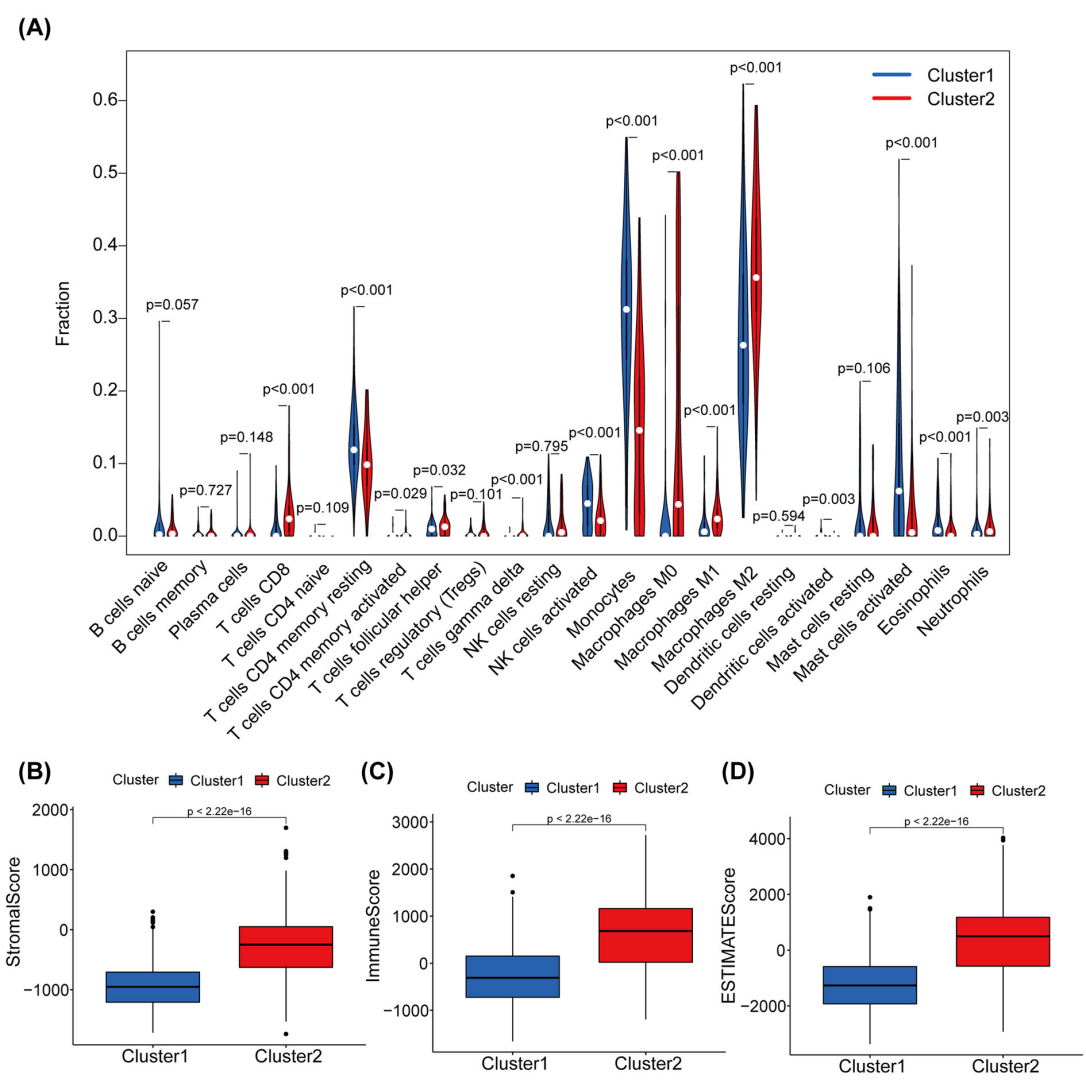
Immune cell infiltration and TME score in cluster 1 and cluster 2 in TCGA (**A**) Infiltration levels of 22 immune cell types in the two clusters in TCGA. (**B–D**) The stromal (B), immune (C), estimate (D) scores in the two clusters in TCGA.

Under the screening condition ‘*P*<0.05’, we explored the differences' percentage of 22 types of immune cells between the two clusters. The results illustrated that cluster 2 had higher infiltrations of Macrophages M2, T cells follicular helper, T cells CD8, and Neutrophils. However, cluster 1 was more tightly linked to activated dendritic cells, activated T cells CD4 memory, activated NK cells, T cells gamma delta, monocytes, activated mast cells, and Eosinophils (Figure 4A). The TME is majorly made up of immune, tumor, and stromal cells, and provides a favorable environment for tumor growth and survival [37]. Therefore, stromal and immune cells (Figure 4B, C) were scored and added as two categories of scores to evaluate the estimate scores. Whereafter, we add the two categories of scores together to evaluate the estimate-scores (Figure 4D). The result showed significant differences in the stromal, immune, and estimate scores between the two clusters. Compared to cluster 1, the stromal, immune, and estimate scores were higher in cluster 2.

**Figure 5:**
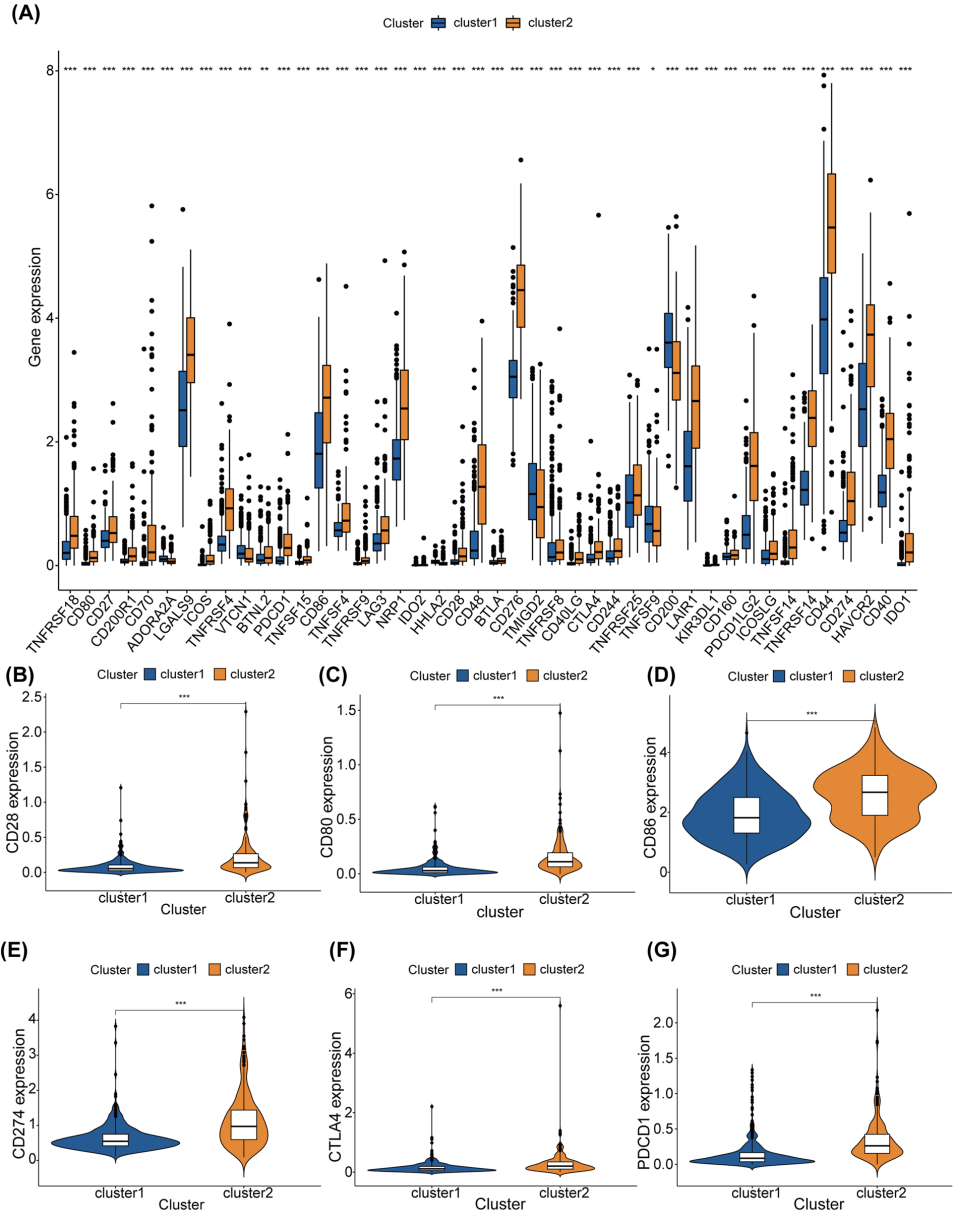
ICPGs differential expression in cluster 1 and cluster 2 in TCGA dataset (**A**) Expression levels of 47 ICPGs in two clusters in TCGA. (**B–G**) The expression levels of CD28 (B), CD80 (C), CD86 (D), CD274 (E), CTLA4 (F), and PDCD1 (G) in the two cluster in TCGA.

Under the screening condition ‘*P*<0.05’, we also explored the distinct ICPGs expression between the two clusters. The results demonstrated that cluster 2 owned higher expression levels of most ICPGs (Figure 5A). Significantly, cluster 2 was more closely linked to CD28, CD80, CD86, CD274, CTLA4, and PDCD1 (Figure 5B-G). However, cluster 1 was more tightly interrelated with ADORA2A, VTCN1, HHLA2, TMIGD2, and CD200 (Figure 5A).

### Construction and confirmation of a prognostic risk model of m6A-associated lncRNAs

**Figure 6:**
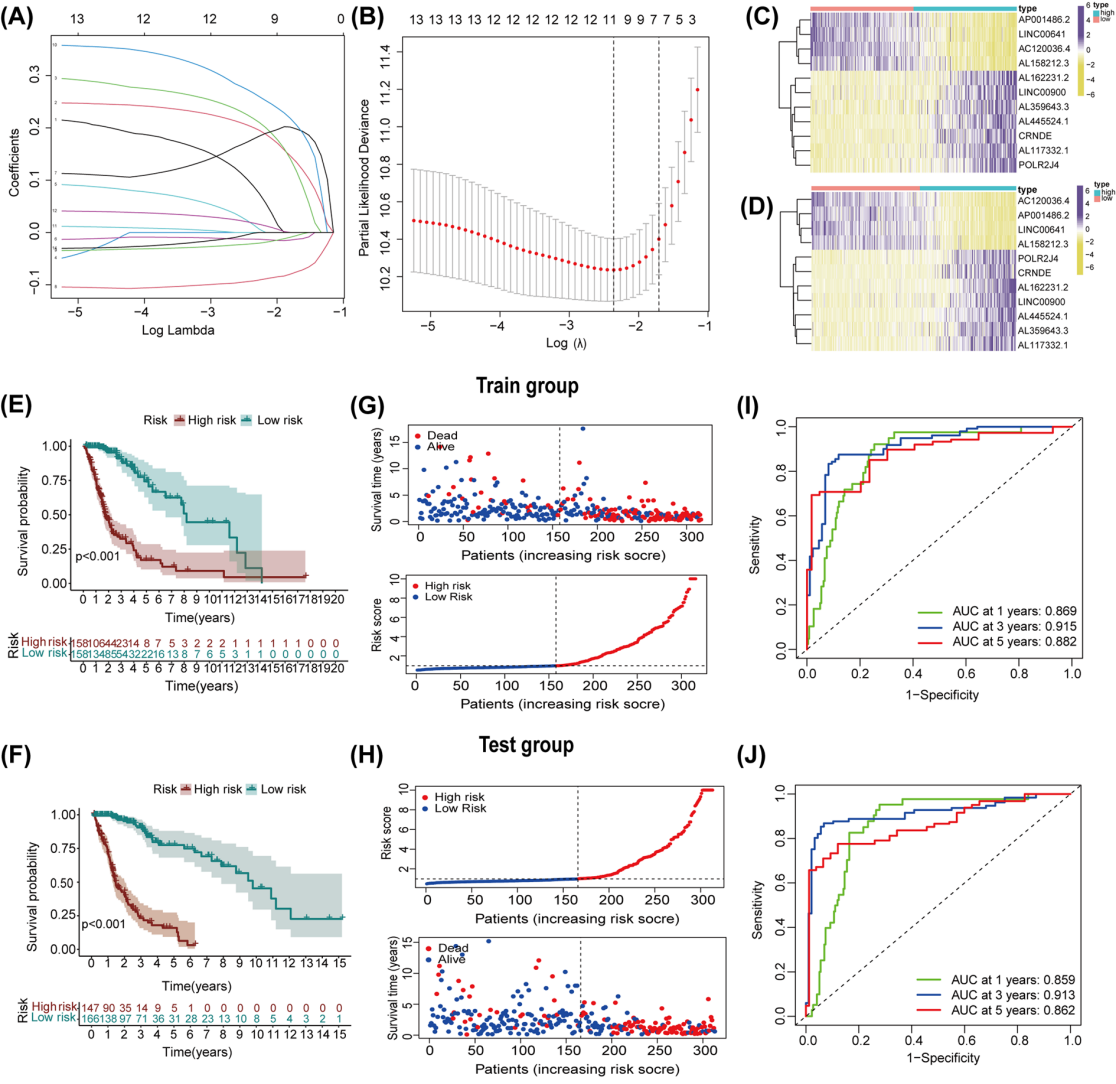
Establishment and verification of prognostic signatures of m6A-connected lncRNAs in TCGA (**A,B**) LASSO regression analysis for quantifying the minimum criteria. (**C,D**) Heatmap revealed the allocation of eleven prognostic signatures in train (C) and test (D) subtypes. (**E,F**) Kaplan-Meier analysis of OS for glioma patients in line with the risk score in train (E) and test (F) subtypes. (**G,H**) Distribution of risk score, OS, and OS status of the eleven prognostic biomarkers in train (G) and test (H) subtypes. (**I,J)** ROC curves reflecting the predictive ability of the risk score in train (I) and test (J) subtypes.

We evaluated the veracity of m6A-associated in forecasting prognosis of glioma patients. The 629 glioma patients were grouped into two subtypes: the train subtype (*n*=316) and the test subtype (*n*=313). We conducted a LASSO regression analysis in line with the expression level of 14 m6A-associated lncRNAs in TCGA train group (Figure 6A,B). We identified eleven important m6A-related lncRNAs, including AL359643.3, AL445524.1, AL62231.2, AL117332.1, AP001486.2, POLR2J4, AC120036.4, LINC00641, LINC00900, CRNDE, and AL158212.3. We measured the risk scores of the train and the test subtypes by conducting the following formula: risk score = (0.0890320658132891 ∗ AL359643.3 expression level) + (0.200759828851677 ∗AL445524.1 expression level) + (0.218041969514275 ∗ AL162231.2 expression level) + (0.0149652762045644 ∗ AL117332.1 expression level) + (-0.0127693323143433 ∗ AP001486.2 expression level) + (0.178557325078596 ∗ POLR2J4 expression level) + (-0.0928092210722127 ∗ AC120036.4 expression level) + (-0.0215325772416436 ∗ LINC00641 expression level) + (0.266934202962373 ∗ LINC00900 expression level) + (0.0180112053612456 ∗ CRNDE expression level) + (-0.00136479047391629 ∗ AL158212.3 expression level). Subsequently, we divided the glioma samples in the train and test subtypes into low-risk and high-risk cohorts in the light of median risk scores. The heatmap revealed that the m6A-related lncRNAs, POLR2J4, CRNDE, AL162231.2, LINC00900, AL445524.1, AL359643.3, and AL117332.1 were elevated in the high-risk cohort. However, AC120036.4, AP001486.2, LINC00641, and AL158212.3 were lower expressed in high-risk cohort (Figure 6C, D).

We analyzed OS between the train and test cohorts in TCGA dataset and found that the OS in low-risk cohort was higher than that in high-risk cohort, regardless of the glioma patients’ subtype (Figure 6E, F). The correlations between OS, OS status, and risk score in train and test subtypes in TCGA dataset are displayed (Figure 6G, H). To confirm the accuracy of the risk model in forecasting the prognosis of glioma patients in TCGA dataset, we explored the time-dependent ROC curves. In the train group, the 1/3/5 years- AUCs were 0.869, 0.915, and 0.882, respectively. Similarly, in the test group, the 1/3/5 years- AUCs were 0.859, 0.913, and 0.862, respectively. (Figure 6I,J). The above results were also confirmed in CGGA cohort (Supplementary Figure S3). Therefore, the risk model may precisely forecast the prognosis of glioma patients.

### Estimation of immune cells’ infiltration and immune checkpoint genes expression with prognostic risk model

Under the screening condition ‘*P*<0.05’, we also inspected the differences’ percentage of 22 types of immune cells between low-risk and high-risk cohorts in train and test subtypes. In train group, we discovered that high-risk cohort had higher infiltrations of Macrophages M2, T cells CD8, B cells naïve, and T cells γ delta. However, low-risk cohort was more closely related to T cells CD4 memory resting, activated NK cells, monocytes, and eosinophils (Supplementary Figure S4A). Compared with low-risk cohort, the stromal, immune, and estimate scores were also higher in high-risk cohort (Supplementary Figure S4B–D). In test group, we also detected that high-risk cohort had higher infiltrations of Macrophages M2, T cells CD8, and NK cells resting. However, low-risk cohort was more closely related to activated NK cells, monocytes, activated mast cells, and Eosinophils (Supplementary Figure S5A). Compared to low-risk cohort, the stromal, immune, and estimate scores were higher in high-risk cohort (Supplementary Figure S5B–D).

Under the screening condition ‘*P*<0.05’, we examined the differences’ expression levels of ICPGs between low-risk and high-risk cohorts in train and test subtypes. The results testified that high-risk cohort owned higher expression levels of most ICPGs in both train and test subtypes (Supplementary Figure S6,7A). Significantly, we perceived that high-risk cohort tend to have higher expression levels of CD28, CD80, CD86, CD274, CTLA4, and PDCD1 (Supplementary Figure S6,7B–G). However, low-risk cohort was more closely related to ADORA2A, VTCN1, HHLA2, TMIGD2, and CD200 (Supplementary Figure S6,7A).

### Risk scores related to clinical features, clusters, and glioma immune scores

**Figure 7:**
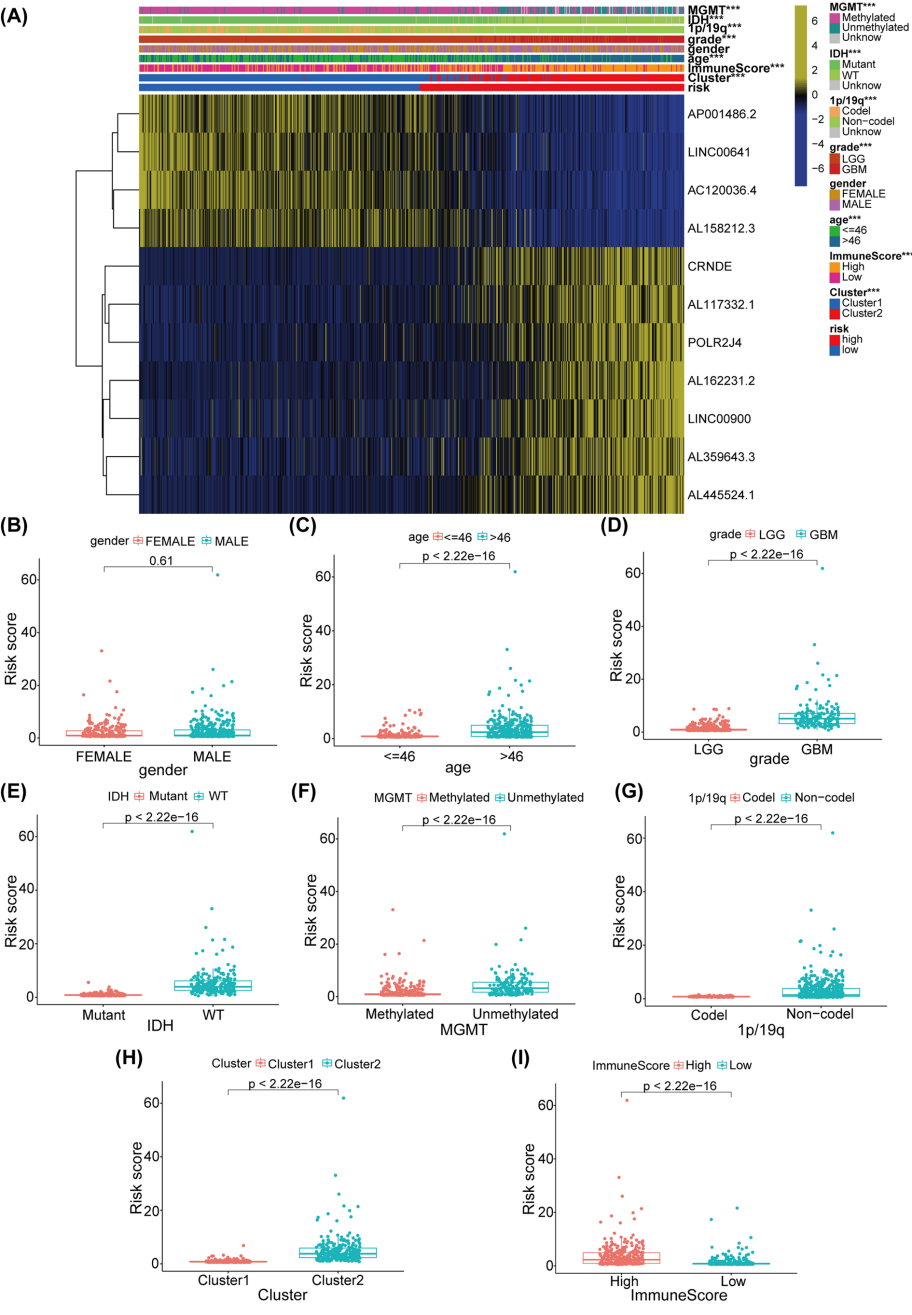
Prognostic risk scores associated with clinical characteristics and immune score in TCGA train sutype (**A**) Heatmap and clinical characteristics of high-risk and low-risk cohorts; *P*<0.001 (***). (**B–I**) The risk scores distributed by gender (B), age (C), grade (D), IDH (E), MGMT (F), 1p/19q (G), cluster l/2 (H), and immune-score (I).

Clinical features, results of cluster analysis, and immune-scores were compared after assembling the information of all glioma samples in low-risk and high-risk cohorts from the train and test train subtypes. The heatmap exhibited that the m6A-associated lncRNAs expression, AL359643.3, AL445524.1, AL162231.2, AL117332.1, POLR2J4, LINC00900, and CRNDE, in high-risk cohort were higher than those in low-risk cohort. However, AP001486.2, AC120036.4, LINC00641, and AL158212.3 expression in high-risk cohort were lower than low-risk cohort (Figure 7A). Beyond gender (Figure 7B), higher risk scores were observed for Age >46 (Figure 7C), Grade 4 (Figure 7D), IDH-WT (Figure 7E), MGMT-unmethylated (Figure 7F), 1p/19q-non-codel (Figure 7G), cluster 2 (Figure 7H), and high immune-scores (Figure 7I). Additionally, the association between identified m6A-associated lncRNAs expression and clinical features was detected (Supplementary Figure S8,9). Subsequently, we explored the OS differences in the two risk cohorts, including age, gender, grade, IDH, MGMT, and 1p/19q. The results affirmed that all OS were higher in low-risk cohort than those in high-risk cohort, except for the grade 4 and 1p/19q-codel cohorts in TCGA dataset (Supplementary Figure S10). Similar results were also examined in CGGA dataset (Supplementary Figure S11). Therefore, our constructed risk model was significative.

To confirm whether clinical features, such as age, gender, grade, IDH, MGMT, and 1p/19q, and risk score, were independent prognostic biomarkers, we carried out Cox analysis for the OS in train and test subtypes. Using univariate Cox analysis, age, gender, grade, IDH, and risk score were regarded as independent prognostic biomarkers for glioma patients in train and test subtypes (Supplementary Figure S12A, C). After further multivariate analysis, age, gender, grade, IDH, and risk score were also found to be independent prognostic biomarkers for glioma in the train and test subtypes (Supplementary Figure S12B, D). Analogous results were also inspected in CGGA dataset (Supplementary Figure S12E-H). In summary, risk scores may play a vital part in forecasting the prognosis of glioma patients.

### Connection between m6A-associated lncRNAs and immune cells' infiltration

**Figure 8:**
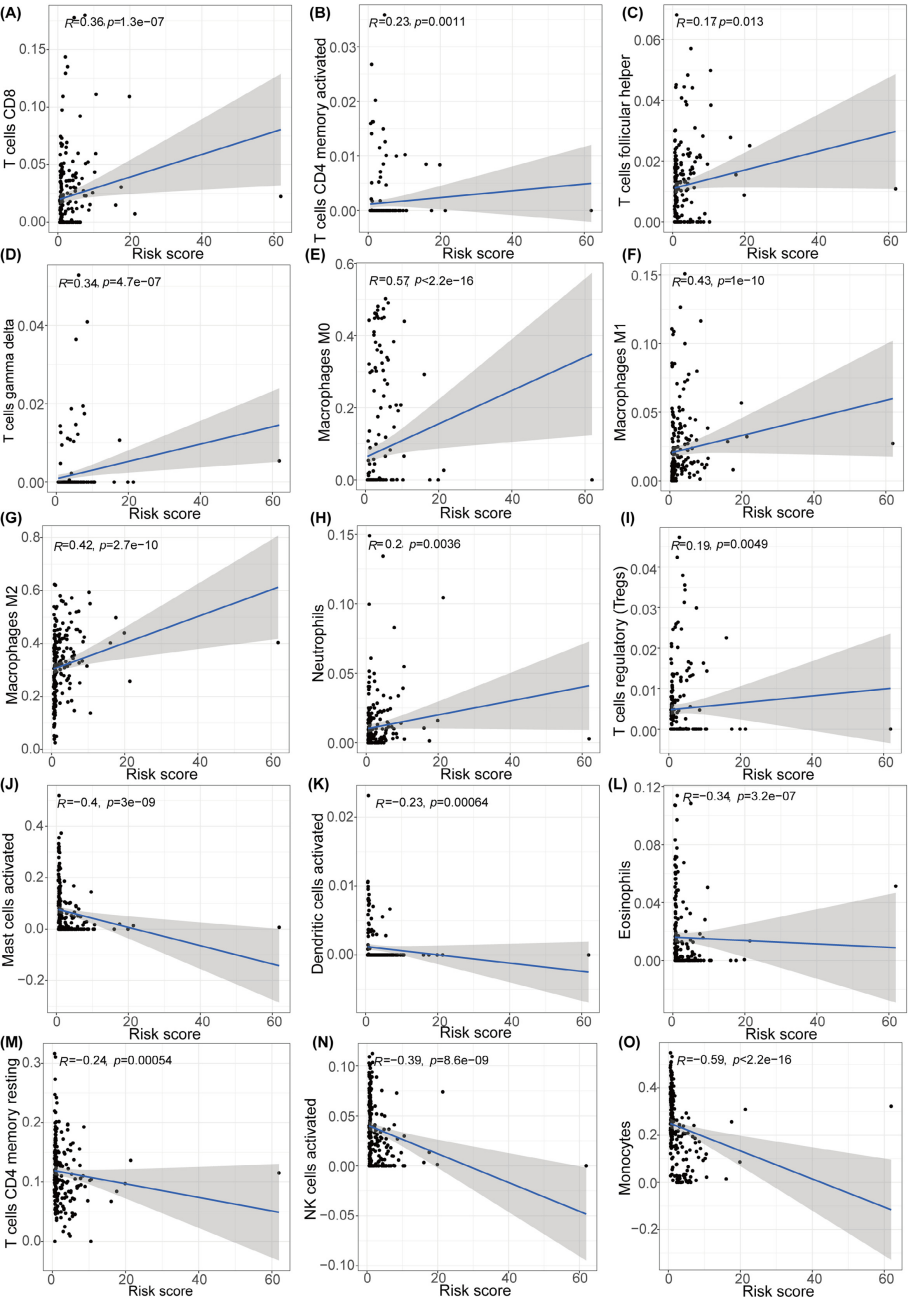
Correlations between risk score and infiltration abundances of 15 immune cell types (**A–O**) T cells CD8 (A), T cells CD4 memory activated (B), T cells follicular helper (C), T cells gamma delta (D), Macrophages M0 (E), Macrophages M1 (F), Macrophages M2 (G), Neutrophils (H), T cells regulatory (Tregs) (I), Mast cells activated (J), Dendritic cells activated (K), Eosinophils (L), T cells CD4 memory resting (M), NK cells activated (N), and Monocytes (O).

We associated risk score with glioma infiltration of fifteen types of immune cells to examine the influence of the eleven m6A-associated lncRNAs on glioma microenvironment. The risk score showed positive correlation with infiltration of T cells CD8 (*P*<0.001) (Figure 8A), T cells CD4 memory activated (*P*<0.001) (Figure 8B), T cells follicular helper (*P*<0.05) (Figure 8C), T cells gamma delta (*P*<0.001) (Figure 8D), macrophages M0 (*P*<0.001) (Figure 8E), macrophages M1 (*P*<0.001) (Figure 8F), macrophages M2 (*P*<0.001) (Figure 8G), and neutrophils (*P*<0.01) (Figure 8H), T cells regulatory (Tregs) (*P*<0.01) (Figure 8I). Conversely, the risk score was inversely interrelated with the infiltration of Mast cells activated (*P*<0.001) (Figure 8J), dendritic cells activated (*P*<0.001) (Figure 8K), and eosinophils (*P*<0.001) (Figure 8L), T cells CD4 memory resting (*P*<0.001) (Figure 8M), NK cells activated (*P*<0.001) (Figure 8N), and monocytes (*P*<0.001) (Figure 8O). This result suggested that the risk signature in line with m6A-connected lncRNAs may be interrelated with the glioma immune microenvironment, which could promote the development of individual treatment strategies and motivate the diversification of therapeutic methods.

### *In vitro* experiments of m6A-associated lncRNAs

**Figure 9:**
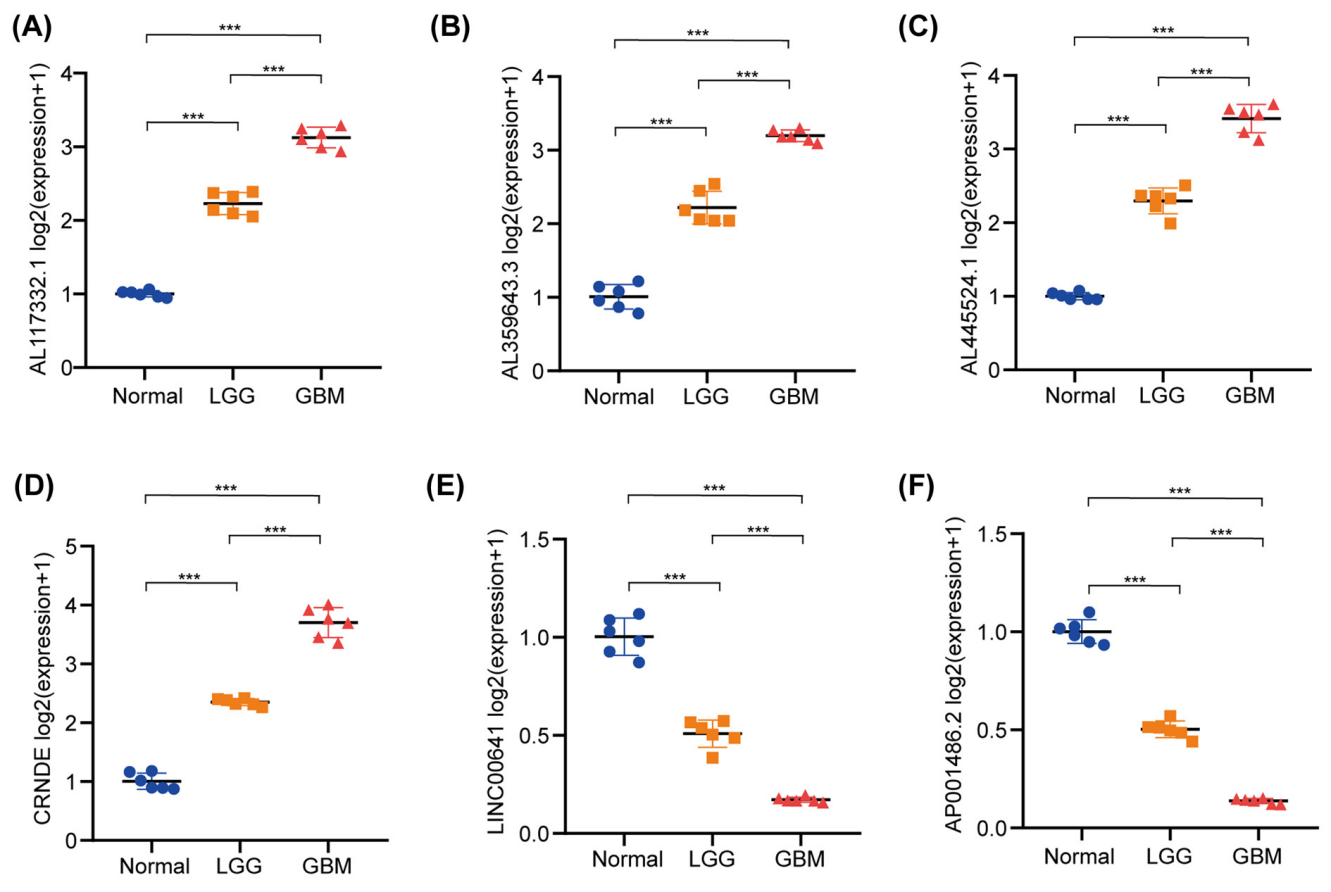
Validation of the expression levels of m6A-associated lncRNAs between normal brain tissues, LGG, GBM tissues by qRT-PCR (**A–D**) The expression levels of AL117332.1 (A), AL359643.3 (B), AL445524.1 (C), CRNDE (D) were much higher in the LGG and GBM tissues when compared to normal brain tissues. (**E,F**) The expression levels of LINC00641 (E) and AP001486.2 (F) in normal brain tissues were much higher than in the LGG and GBM tissues.

The expression levels of AL117332.1, AL359643.3, AL445524.1, CRNDE were much higher in the LGG and GBM tissues when compared with normal brain tissues by qRT-PCR (Figure 9A–D) and immunofluorescence (Supplementary Figure S13A–D). However, the expression levels of LINC00641 and AP001486.2 in normal brain tissues were much higher than in the LGG and GBM tissues qRT-PCR (Figure 9E,F) and immunofluorescence (Supplementary Figure S13E, F). Additionally, we also detected that the expression levels of AL117332.1, AL359643.3, AL445524.1, CRNDE were much higher in the IDH-WT and 1p/19q-non-codel subgroups when compared to IDH-mutant and 1p/19q-codel subgroups (Supplementary Figure S14A-D). However, the expression levels of LINC00641 and AP001486.2 in the IDH-mutant and 1p/19q-codel subgroups were much higher than in the IDH-WT and 1p/19q-non-codel subgroups (Supplementary Figure S14E, F).

## Discussion

LncRNAs are non-coding RNAs that can be modified by m6A methylation in cancers [38]. Increasing evidence has demonstrated that m6A-modified lncRNAs are tightly linked to immune cell’ infiltration of tumors [29,39–42]. However, there have been no reports about immune infiltration of m6A-connected lncRNAs in glioma. Thus, it is essential to have an in-depth understanding of the correlation between m6A-associated lncRNAs and glioma immune microenvironment.

We collected data from the TCGA that included 629 glioma patients to inspect the roles of m6A-connected lncRNAs on the TME. We selected 23 m6A genes that were reported in previous research and explored their mutations using TCGA datasets. Furthermore, we established a co-expression network which reflected the conjunction between the 23 m6A genes and lncRNAs by analyzing the genes' expression files. Using univariate Cox analyses, we found that 14 lncRNAs were underlying prognostic biomarkers of glioma and that most of them were highly expressed in GBM samples when compared with those in LGG samples. Using consensus clustering, the glioma samples were classified into two clusters in the light of the m6A-associated lncRNAs expression. Additionally, the OS in cluster 1 was significantly higher than that in cluster 2. M6A-related lncRNAs, including AL162231.2, LINC00900, LBX2-AS1, AL445524.1, AL117332.1, AL138724.2, AL359643.3, POLR2J4, AC026401.3, and CRNDE had higher expression levels in cluster 2 than those in cluster 1. Conversely, the expression levels of AC120036.4, AL158212.3, AP001486.2, and LINC00641 in cluster 2, were lower than those in cluster 1. Moreover, the clinical features including MGMT, IDH, 1p/19q, grade, and age were tightly linked to our cluster analysis. Interestingly, the cluster 2 had higher infiltrations of Macrophages M2, T cells follicular helper, T cells CD8, and Neutrophils. However, cluster 1 had higher infiltrations of activated dendritic cells, activated T cells CD4 memory, activated NK cells, T cells gamma delta, monocytes, activated mast cells, and Eosinophils. In addition, cluster 2 possessed higher expression levels of CD28, CD80, CD86, CD274, CTLA4, and PDCD1. However, cluster 1 owned higher expression levels of ADORA2A, VTCN1, HHLA2, TMIGD2, and CD200. We next investigated differences in tumor-related pathways between the two clusters using multiple GSEA and found that the major enrichment pathways of cluster 2 were ‘cytokine–cytokine receptor interaction’, ‘nucleotide excision repair’, ‘antigen processing and presentation’, ‘cell cycle’, ‘primary immunodeficiency’, and ‘p53 signaling pathway’. However, the main enrichment pathways of cluster 1 were ‘long-term depression’, ‘inositol phosphate metabolism’, and ‘WNT signaling pathway’.

The prognostic signatures of m6A-connected lncRNAs in glioma patients were evaluated by constructing a risk model. Patients were grouped into low-risk and high-risk subtypes in line with the risk score that was calculated by our formula. Eleven m6A-associated lncRNAs were employed to validate the risk signature by conducting the LASSO regression analysis. Whether the patients were in the train or test subtype, the OS of low-risk subtype was apparently higher than that in high-risk subtype. At 1, 3 and 5 years, AUCs were0.869, 0.915, and 0.882 in the train subtype and 0.859, 0.913, and 0.862 in the test subtype, respectively. Additionally, the results of Cox regression analyses proved that age, gender, grade, IDH and risk score were independent prognostic biomarkers for glioma patients in both train and test subtypes. The clinical features, results of cluster analysis, and immune-scores were also analyzed in low-risk and high-risk cohorts from the train and test subtypes. In train group, high-risk cohort had higher infiltrations of macrophages M2, T cells CD8, B cells naïve, and T cells gamma delta. However, low-risk cohort was more closely related to T cells CD4 memory resting, activated NK cells, monocytes, and eosinophils. In test group, high-risk cohort had higher infiltrations of Macrophages M2, T cells CD8, and NK cells resting. However, low-risk cohort had higher infiltrations of activated NK cells, monocytes, activated mast cells, and eosinophils. Both in train and test group, high-risk cohort had higher expression levels of CD28, CD80, CD86, CD274, CTLA4, and PDCD1. However, low-risk cohort was more closely related to ADORA2A, VTCN1, HHLA2, TMIGD2, and CD200. In a word, m6A-connected lncRNAs were tightly interrelated with the infiltrations of diverse immune cells, demonstrating that the risk model might forecast the glioma immune microenvironment.

Eventually, we testified that the m6A-connected lncRNAs was abnormally expressed in glioma by conducting *in vitro* experiments. However, there were several limitations in this research. More independent glioma datasets should be implemented to certify the identified prognostic m6A-associated lncRNAs. In addition, the biological functions of the m6A-connected lncRNAs should be inspected utilizing *in vitro* and *in vivo* experiments.

## Conclusion

In summary, our research constructed a m6A-connected lncRNA prognostic signature and evaluated the correlation with glioma immune infiltration. The signature might provide underlying targets for enhancement in immunotherapy for patients with glioma.

## Supplementary Material

Supplementary Figures S1-S14 and Tables S1-S5

## Data Availability

The data analyzed in this study can be found in the TCGA (http://cancergenome.nih.gov/), CGGA (http://www.cgga.org.cn/), and cBioPortal (http://cbioportal.org) websites.

## Abbreviations

ANOVA: analysis of variance
AUC: area under the curve
CIBERSORT: cell type identification by estimating relative subsets of RNA transcripts
ESTIMATE: estimation of stromal and immune cells in malignant tumor tissues using expression data
FDR: false discovery rate
FPKM: fragment per kilobase of exon model per million
GO: Gene Ontology
GSEA: Gene Set Enrichment Analysis
ICPGs: immune-checkpoint genes
KEGG: Kyoto Encyclopedia of Genes and Genomes
LASSO: least absolute shrinkage and selection operator
lncRNA: long non-coding RNA
NES: normalized enrichment score
Nom: Nominal
OS: overall survival
PCA: principal component analysis
ROC: receiver operating characteristic
TCGA: The Cancer Genome Atlas
TME: tumor microenvironment
tSNE: t-distributed stochastic neighbor embedding
