# Supplementary material for: N6-methyladenosine (m6A)-connected lncRNAs are linked to survival and immune infiltration in glioma patients
Source: Biosci Rep. 2023 May 5;43(5):BSR20222100. doi: 10.1042/BSR20222100 (PMC10170299; doi:10.1042/BSR20222100)
Supplement: Supplementary Figures S1-S14 and Tables S1-S5 [file BSR-2022-2100_supp.pdf]

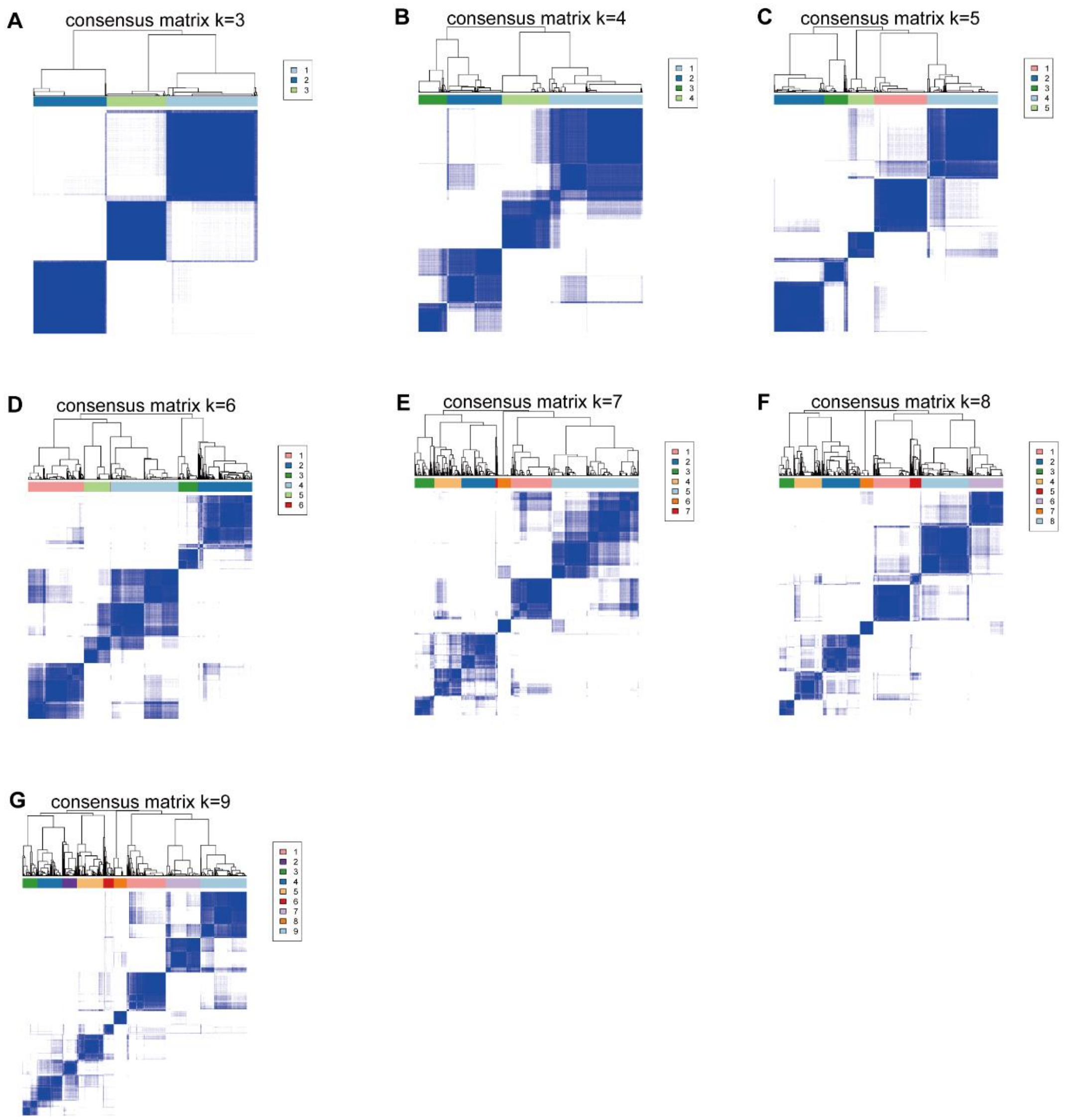

Supplementary Figure S1. Consensus clustering analysis with k from 3 to 9 in CDF. **(A-G)** Glioma TCGA cohort was categorized as clusters with k=3 **(A)**, k=4 **(B)**, k=5 **(C)**, k=6 **(D)**, k=7 **(E)**, k=8 **(F)**, k=9 **(G)**.

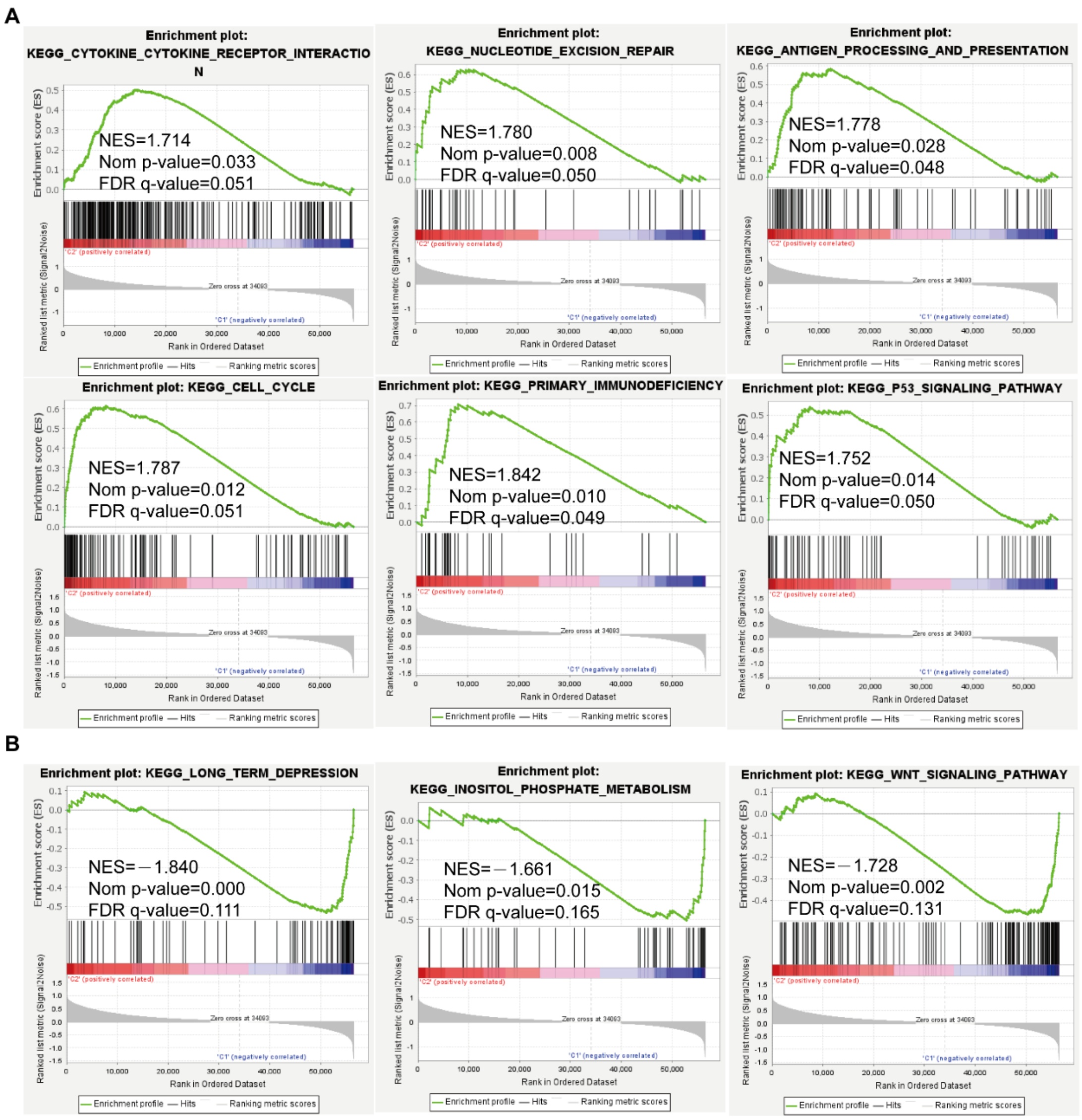

Supplementary Figure S2. GSEA was performed to forecast underlying pathway and functions between cluster 2 and cluster 1. (**A**) The main enrichment pathways of cluster 2. (**B**) The main enrichment pathways of cluster 1.

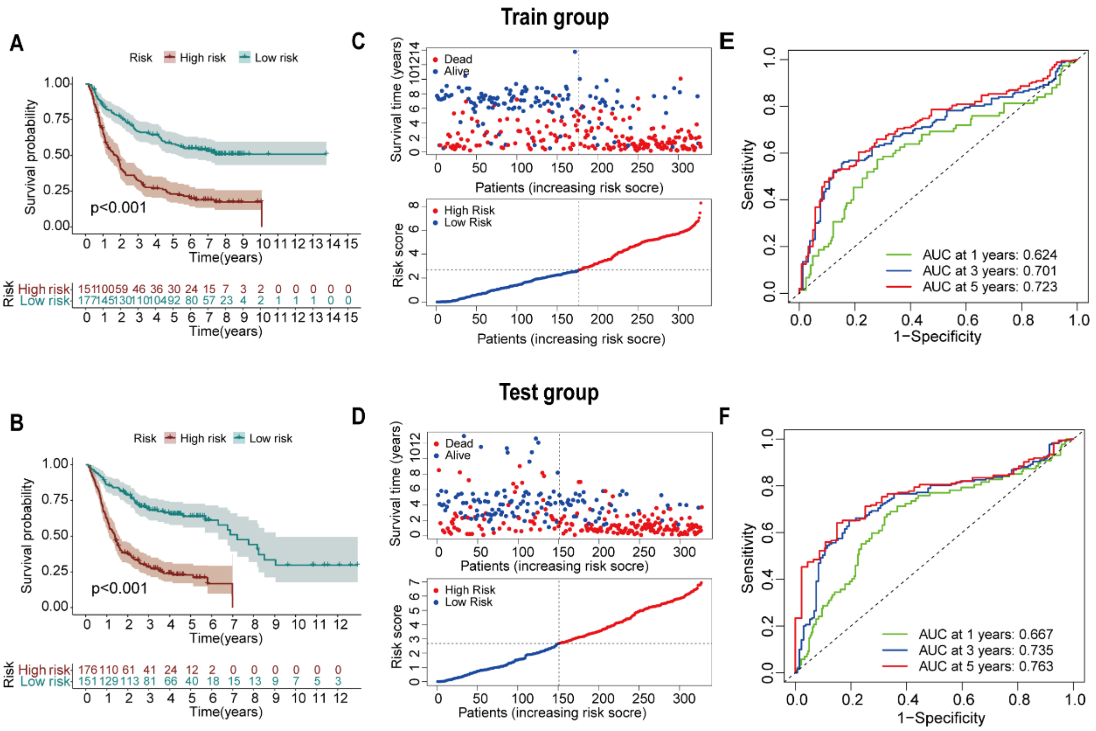

Supplementary Figure S3. Conformation of prognostic signatures of m6A-connected lncRNAs in CGGA. **(A-B**) Kaplan-Meier analysis of OS for glioma patients in line with the risk score in train (**A**) and test (**B**) subtypes. **(C-D**) Distribution of risk score, OS, and OS status of the eleven prognostic biomarkers in train (**C**) and test (**D**) subtypes. (**E-F)** ROC curves reflecting the predictive ability of the risk score in train (**E**) and test (**F**) subtypes.

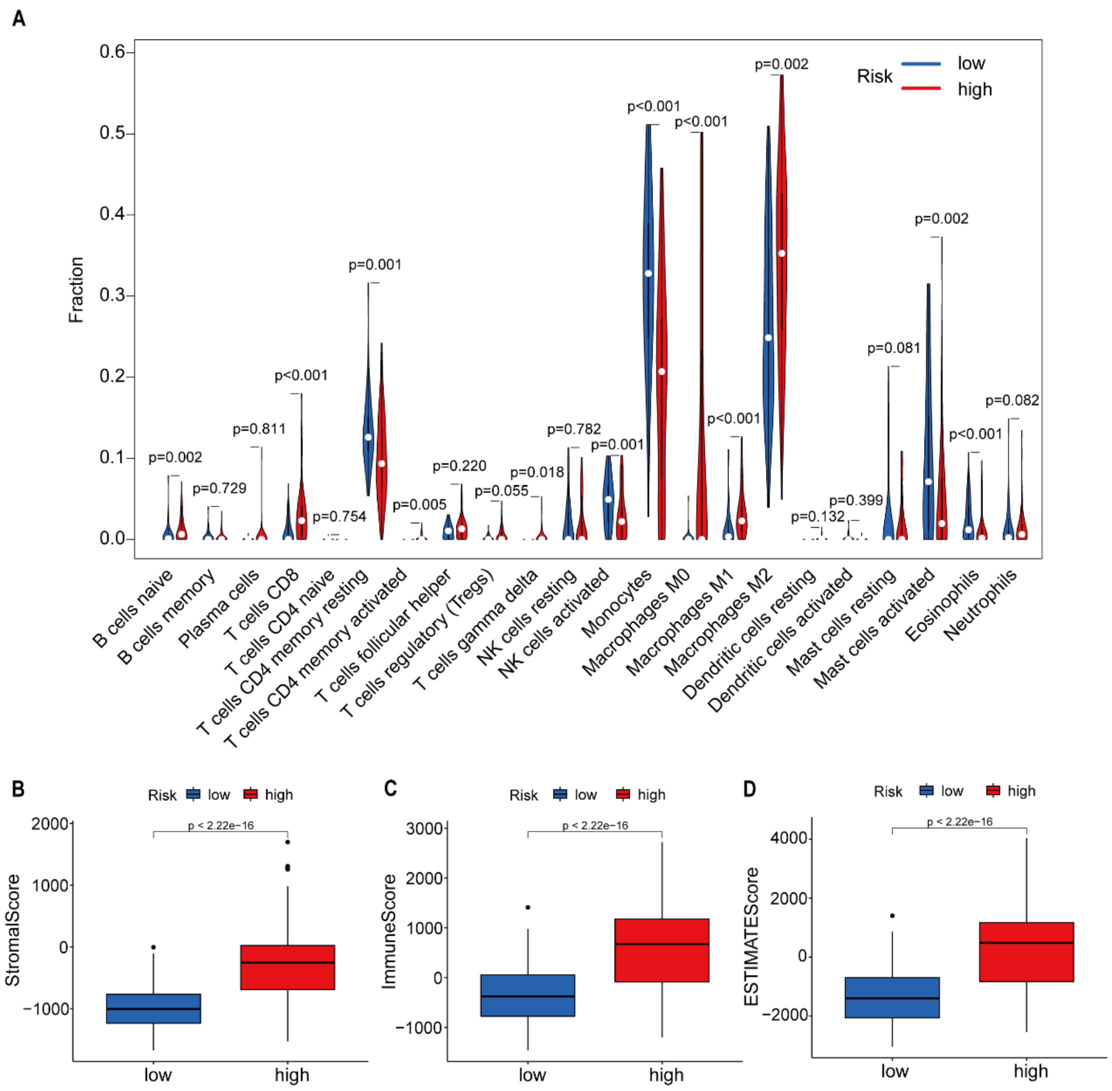

Supplementary Figure S4. Immune cell infiltration and TME score in low-risk and high-risk cohorts in train subtype. (**A**) Infiltration levels of 22 immune cell types in low-risk and high-risk cohorts in train subtype. (**B-D**) The stromal (**B**), immune (**C**), estimate (**D**) scores in low-risk and high-risk cohorts in train subtype.

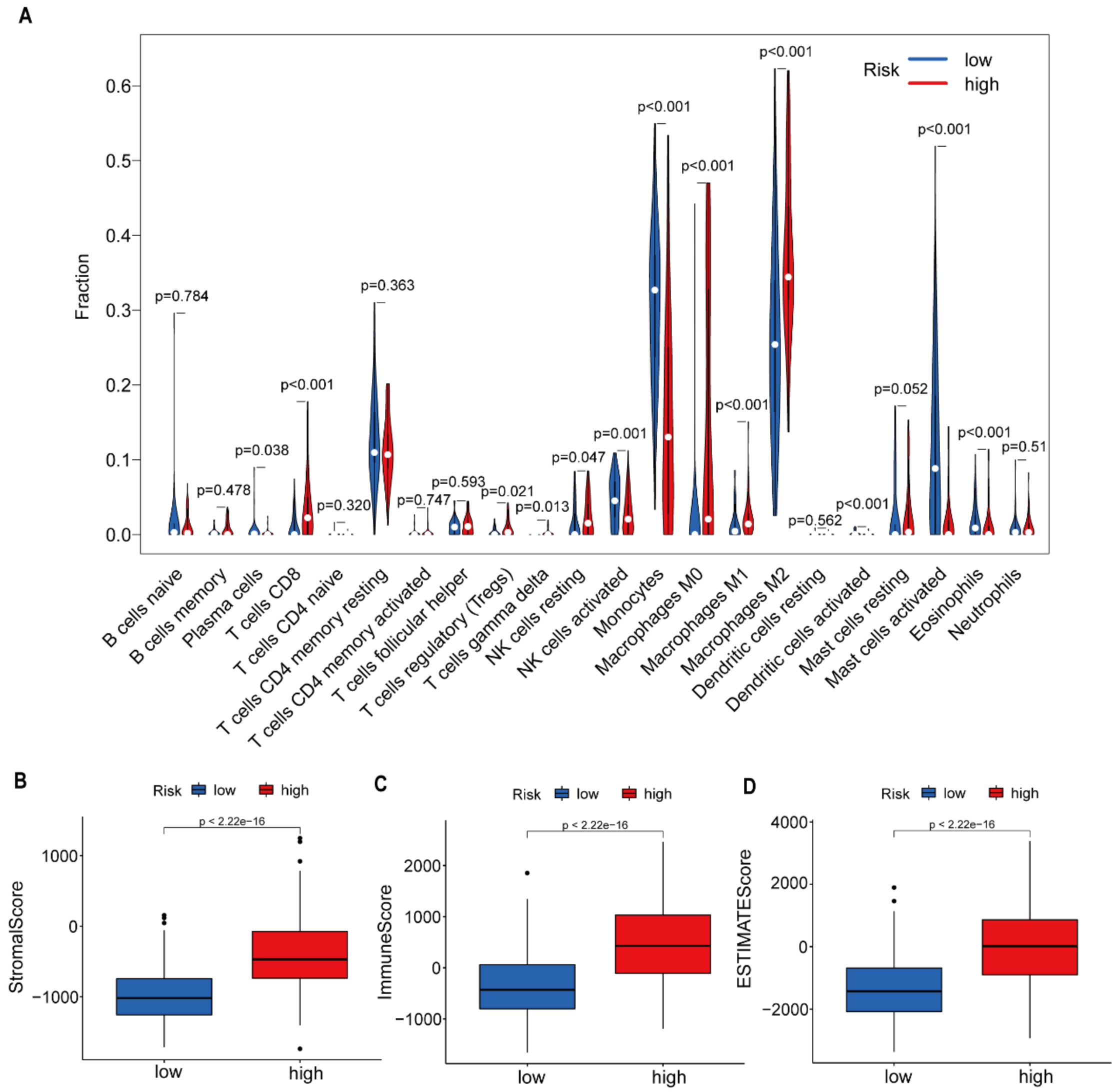

Supplementary Figure S5. Immune cell infiltration and TME score in low-risk and high-risk cohorts in test subtype. (**A**) Infiltration levels of 22 immune cell types in low-risk and high-risk cohorts in test subtype. (**B-D**) The stromal (**B**), immune (**C**), estimate (**D**) scores in low-risk and high-risk cohorts in train subtype.

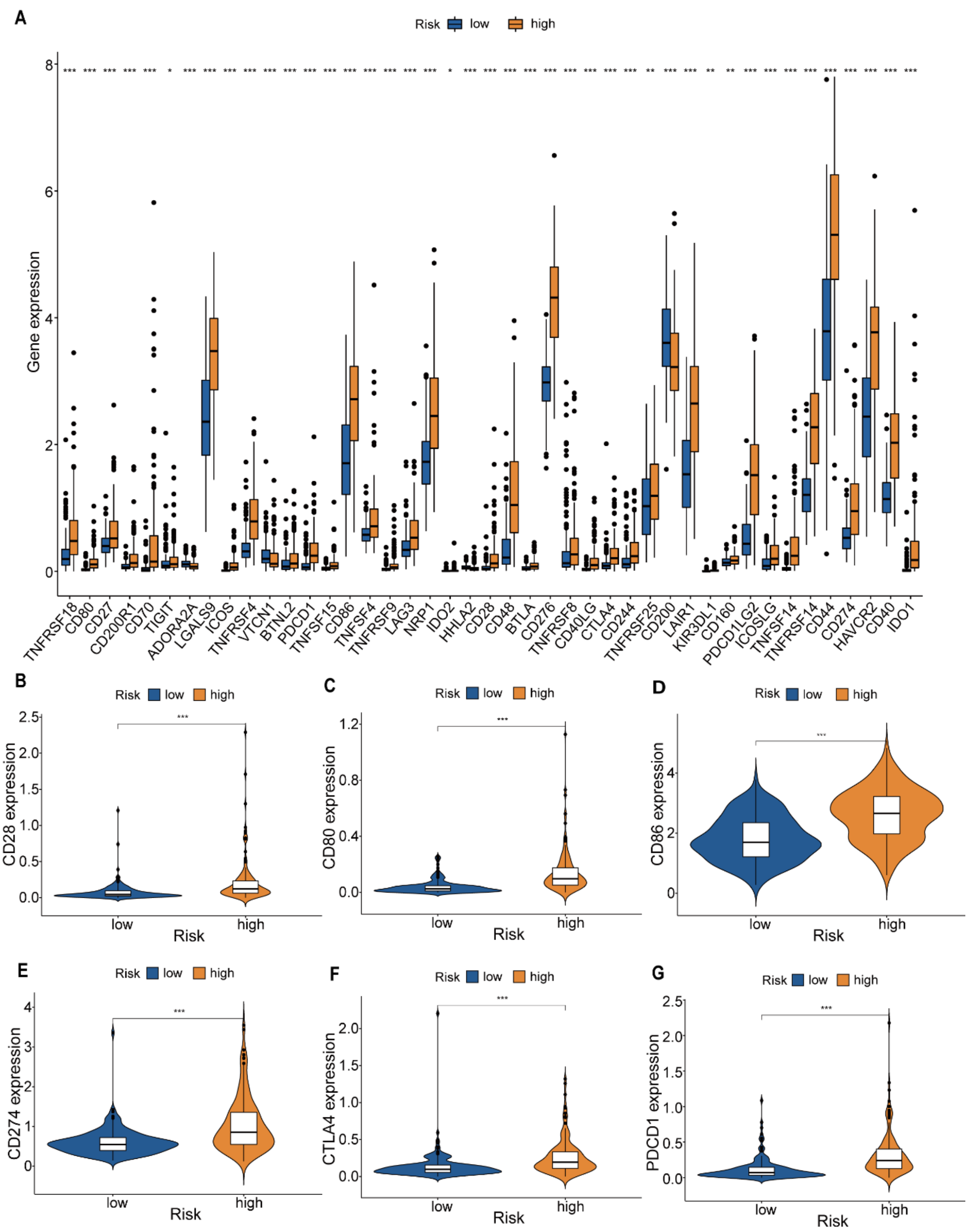

Supplementary Figure S6. ICPGs differential expression in low-risk and high-risk cohorts in train subtype. (**A**) Expression levels of 47 ICPGs in low-risk and high-risk cohorts in train subtype. (**B-G**) The expression levels of CD28 (**B**), CD80 (**C**), CD86 (**D**), CD274 (**E**), CTLA4 (**F**), PDCD1 (**G**) in low-risk and high-risk cohorts in train subtype.

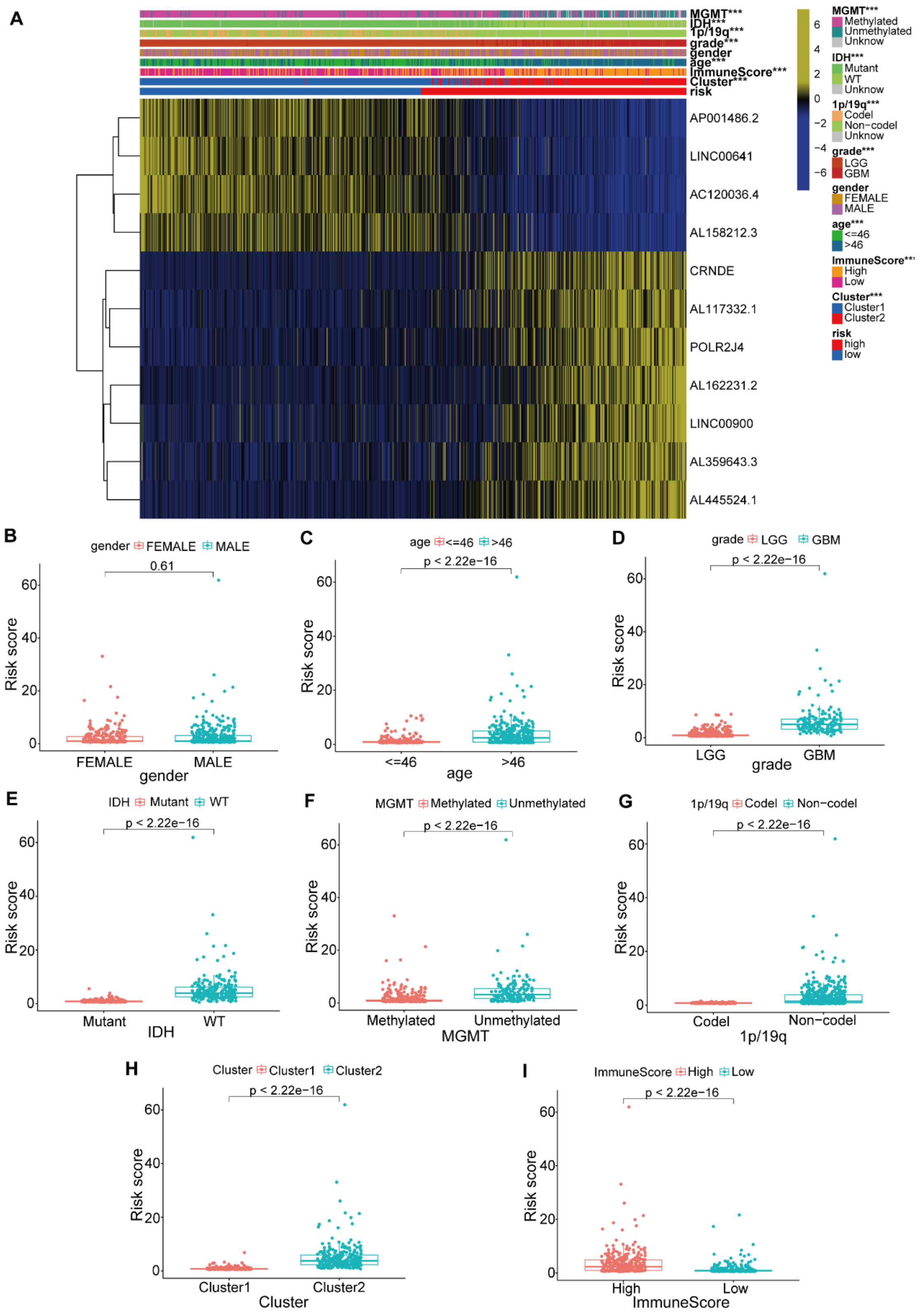

Supplementary Figure S7. ICPGs differential expression in low-risk and high-risk cohorts in test subtype. (**A**) Expression levels of 47 Immune-checkpoint genes in low-risk and high-risk cohorts in test subtype. (**B-G**) The expression levels of CD28 (**B**), CD80 (**C**), CD86 (**D**), CD274 (**E**), CTLA4 (**F**), PDCD1 (**G**) in low-risk and high-risk cohorts in test subtype.

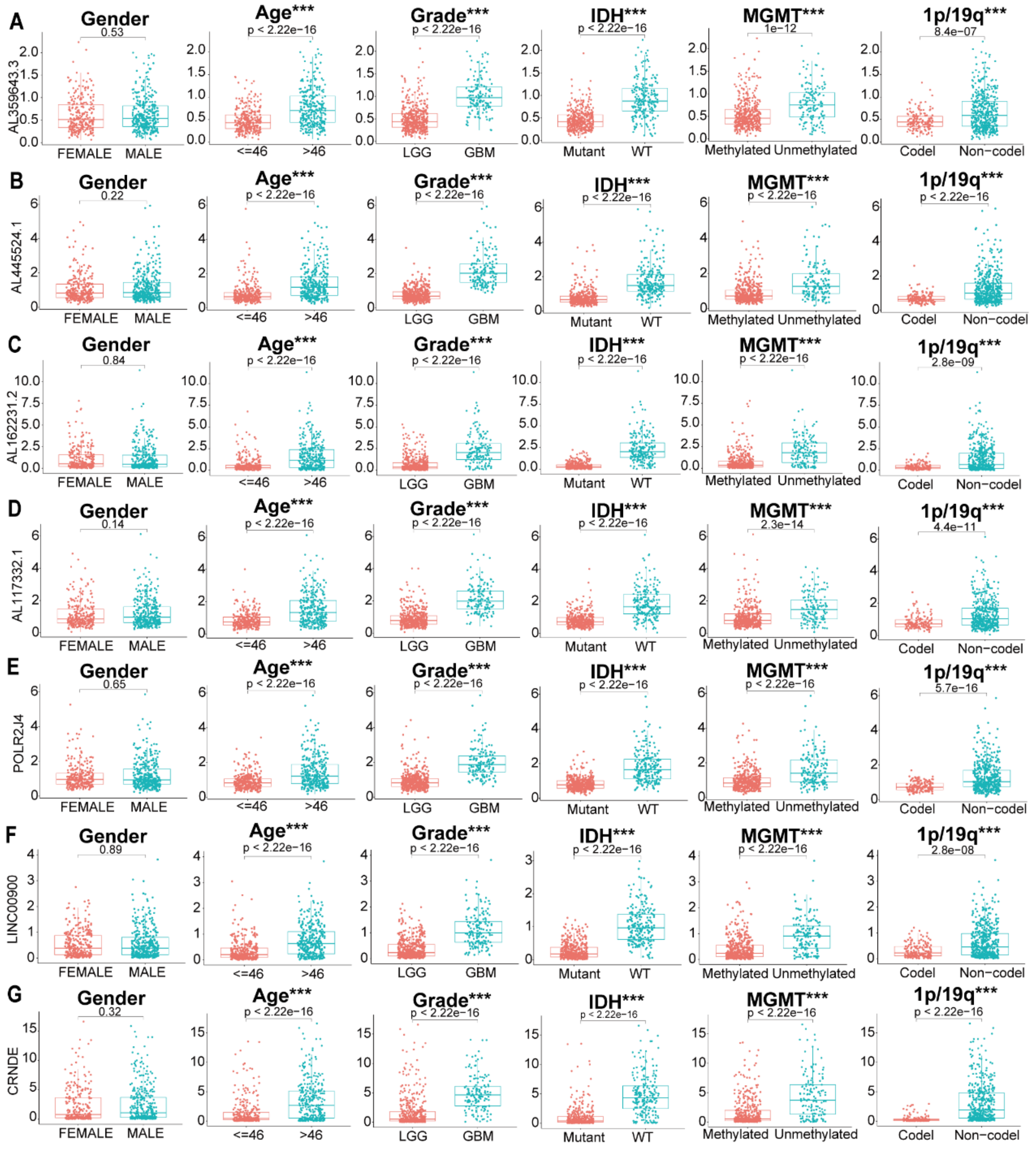

Supplementary Figure S8. Correlation between clinical features and upregulated m6A-connected lncRNAs. AL359643.3 (**A**), AL445524.1 (**B**), AL162231.2 (**C**), AL117332.1 (**D**), POLR2J4 (**E**), LINC00900 (**F**), and CRNDE (**G**).

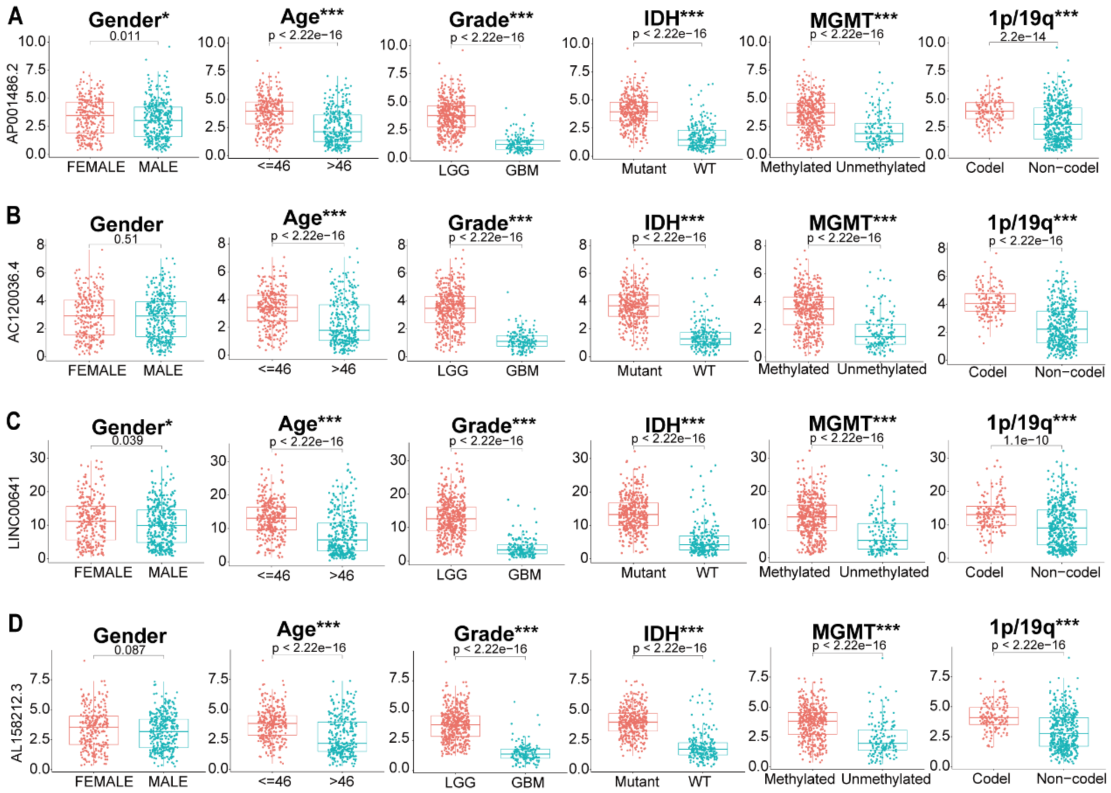

Supplementary Figure S9. Correlation between clinical features and downregulated m6A-connected lncRNAs. AP001486.2 (**A**), AC120036.4 (**B**), LINC00641 (**C**), and AL158212.3 (**D**).

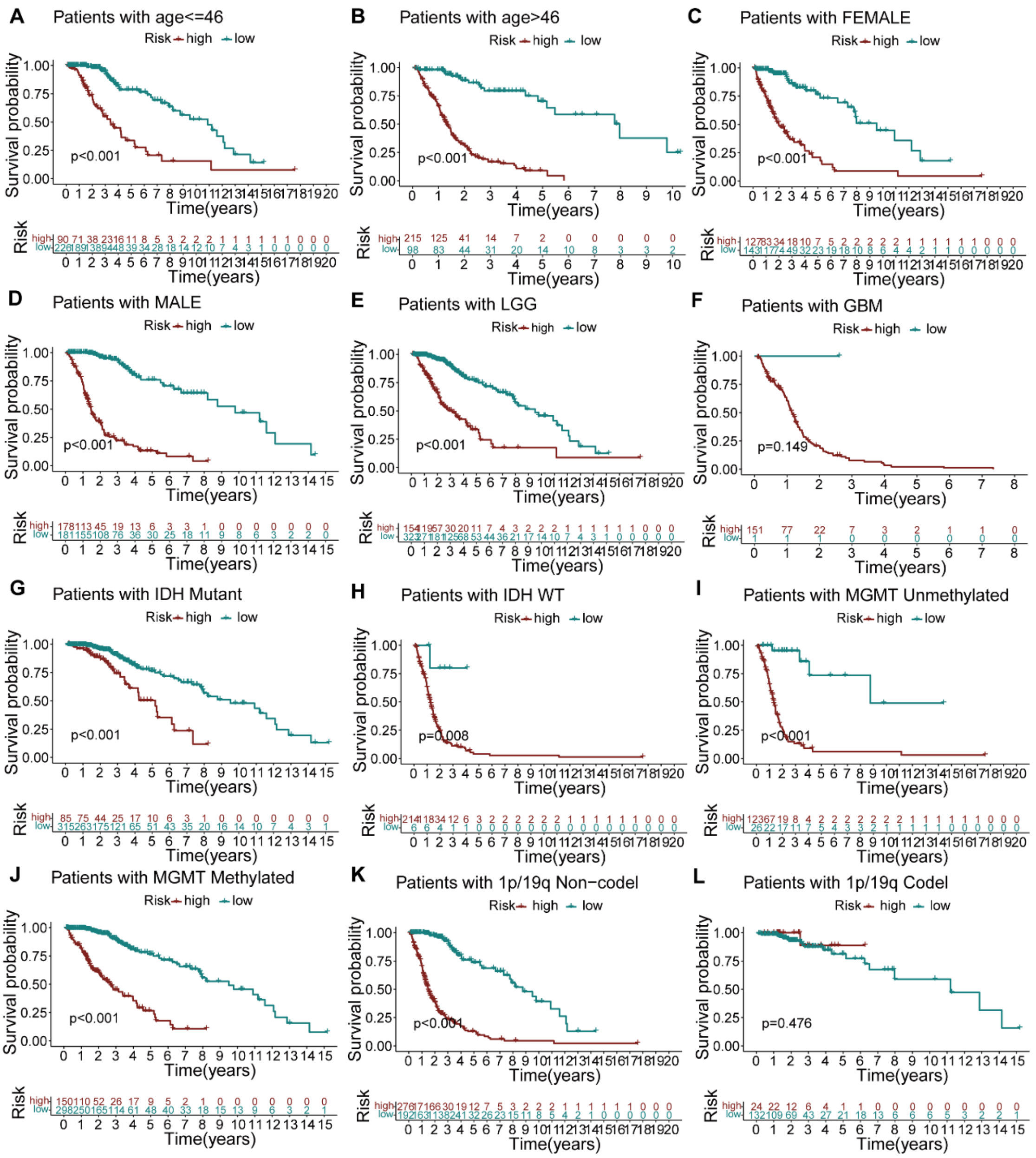

Supplementary Figure S10. Kaplan-Meier analysis demonstrated that distinct OS of differentiated clinical features of glioma patients in high-risk and low-risk cohorts in TCGA. (**A-L**) age<=46 (**A**), age>46 (**B**), FEMALE (**C**), MALE (**D**), LGG (**E**), GBM (**F**), IDH Mutant (**G**), WT (**H**), MGMT Unmethylated (**I**), Methylated (**J**), 1p/19q Non-codel (**K**), and Codel (**L**).

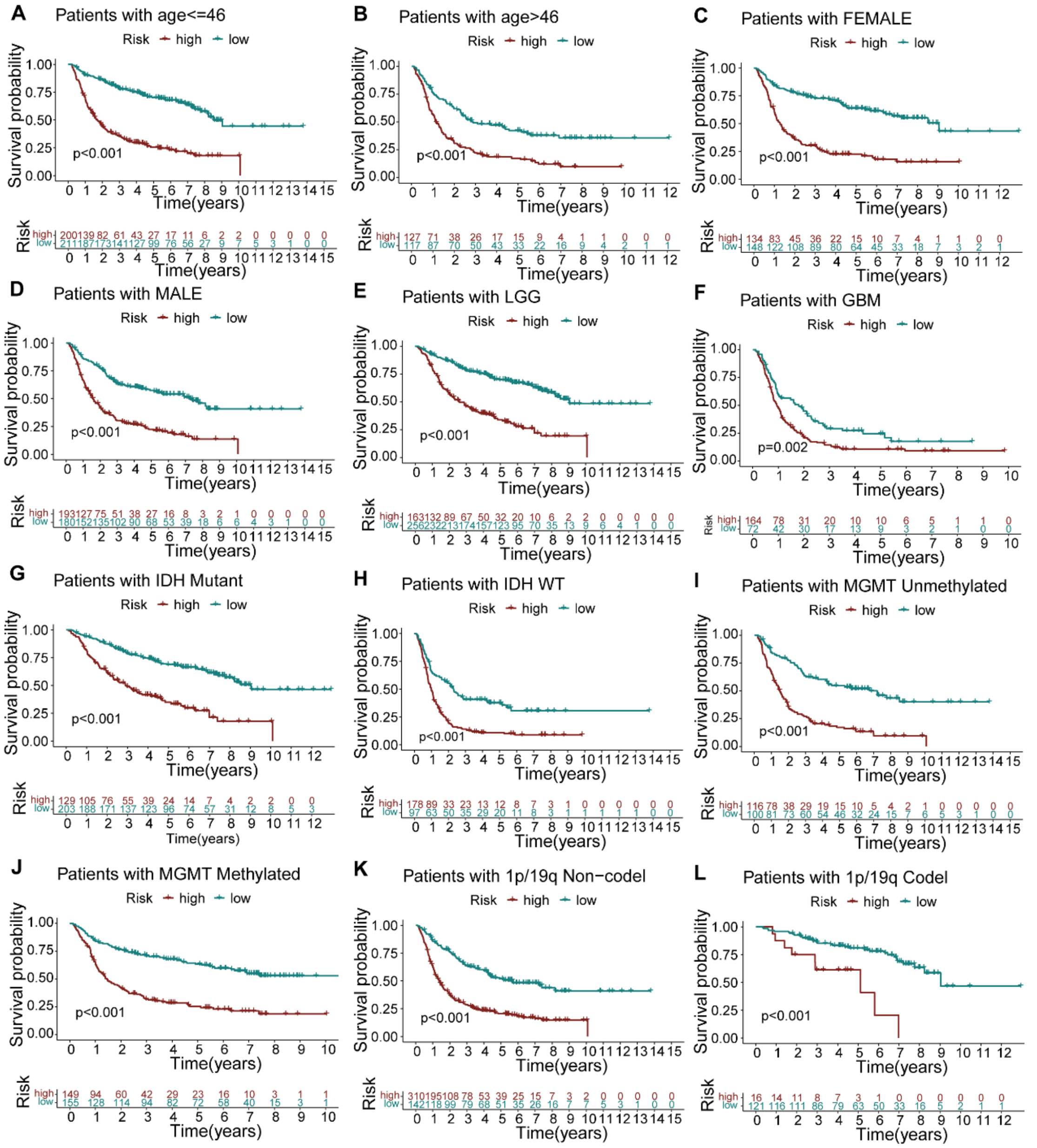

Supplementary Figure S11. Kaplan-Meier analysis demonstrated that distinct OS of differentiated clinical features of glioma patients in high-risk and low-risk cohorts in CGGA. (**A-L**) age<=46 (**A**), age>46 (**B**), FEMALE (**C**), MALE (**D**), LGG (**E**), GBM (**F**), IDH Mutant (**G**), WT (**H**), MGMT Unmethylated (**I**), Methylated (**J**), 1p/19q Non-codel (**K**), and Codel (**L**).

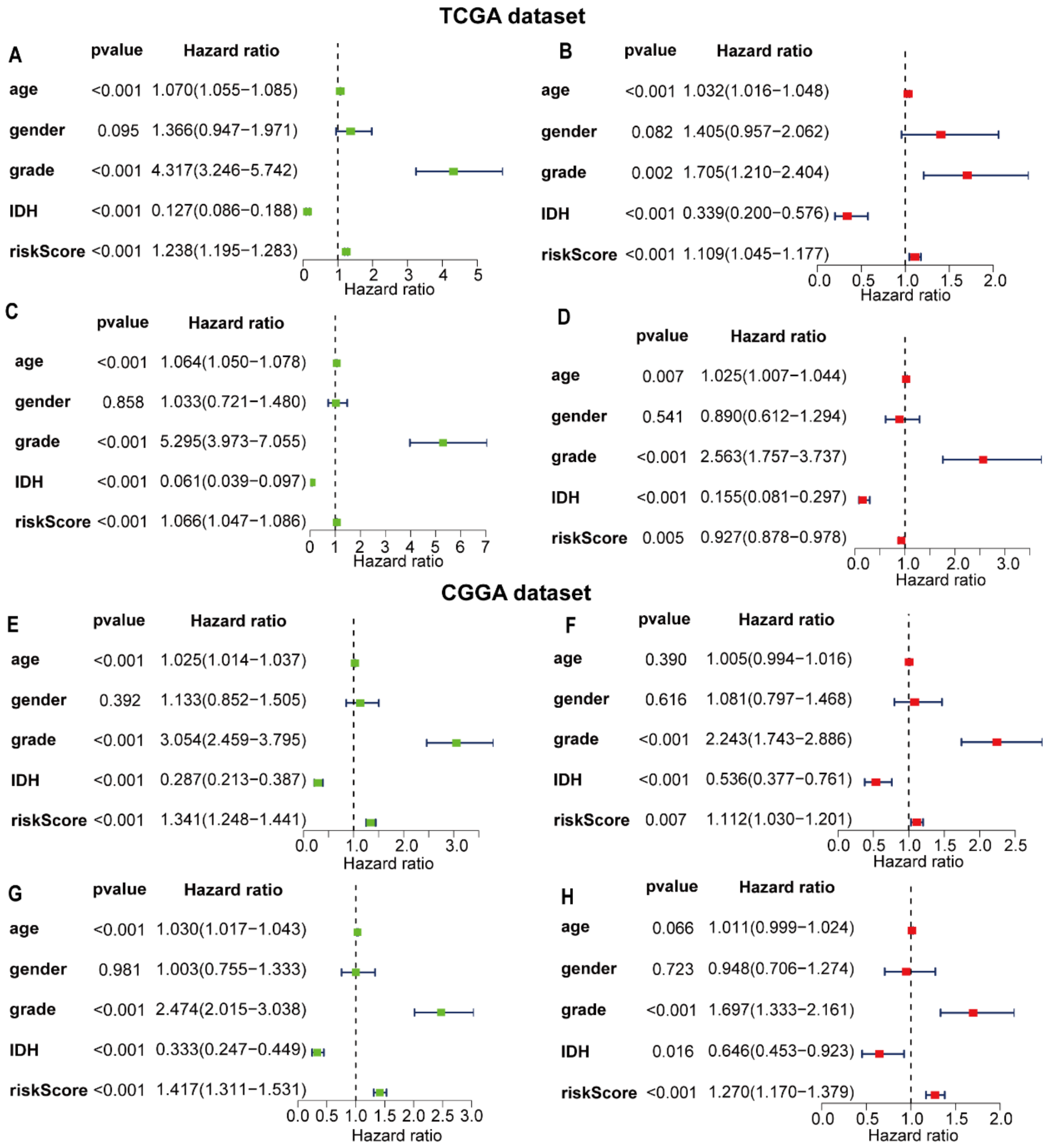

Supplementary Figure S12. Cox regression analysis for the train and test subtypes in TCGA and CGGA cohorts. (**A-B**) Univariate (**A**) and multiple (**B**) Cox regression was executed in the train subtype in TCGA. (**C-D**) Univariate (**C**) and multiple (**D**) Cox regression was executed in the test subtype in TCGA. (**E-F**) Univariate (**E**) and multiple (**F**) Cox regression was executed in the train subtype in CGGA. (**G-H**) Univariate (**G**) and multiple (**H**) Cox regression was executed in the test subtype in CGGA.

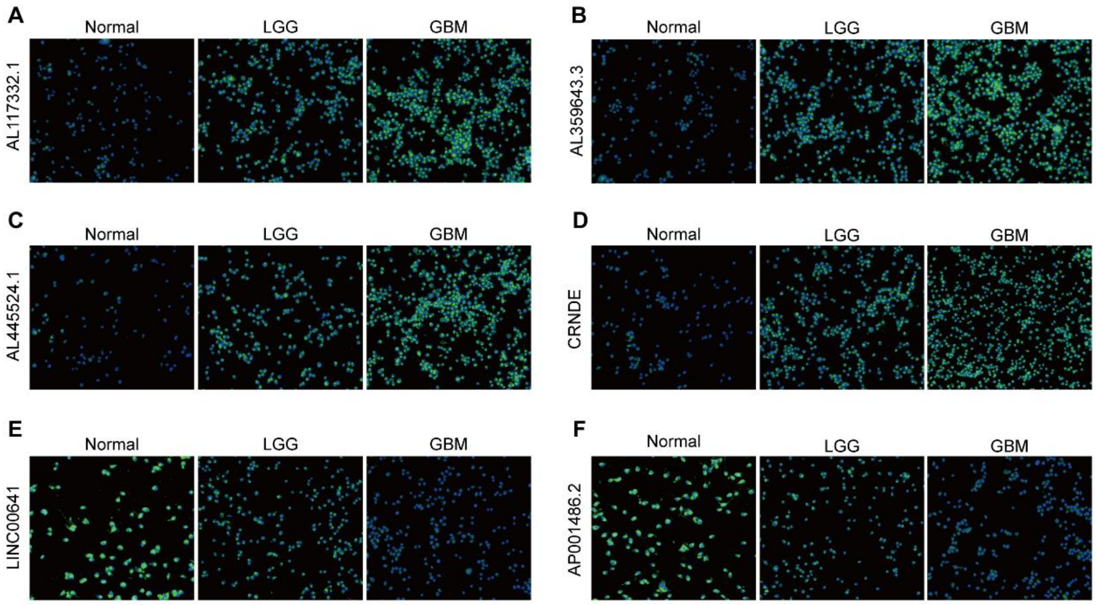

Supplementary Figure S13. Confirmation of the expression levels of m6A-associated lncRNAs between normal brain tissues, LGG, GBM tissues by immunofluorescence. (**A-D**) The expression levels of AL1177332.1 (**A**), AL359643.3 (**B**), AL445524.1 (**C**), CRNDE (**D**) were much higher in the LGG and GBM tissues when compared to normal brain tissues. **(E-F**) The expression levels of LINC00641 (**E**) and AP001486.2 (**F**) in normal brain tissues were much higher than in the LGG and GBM tissues.

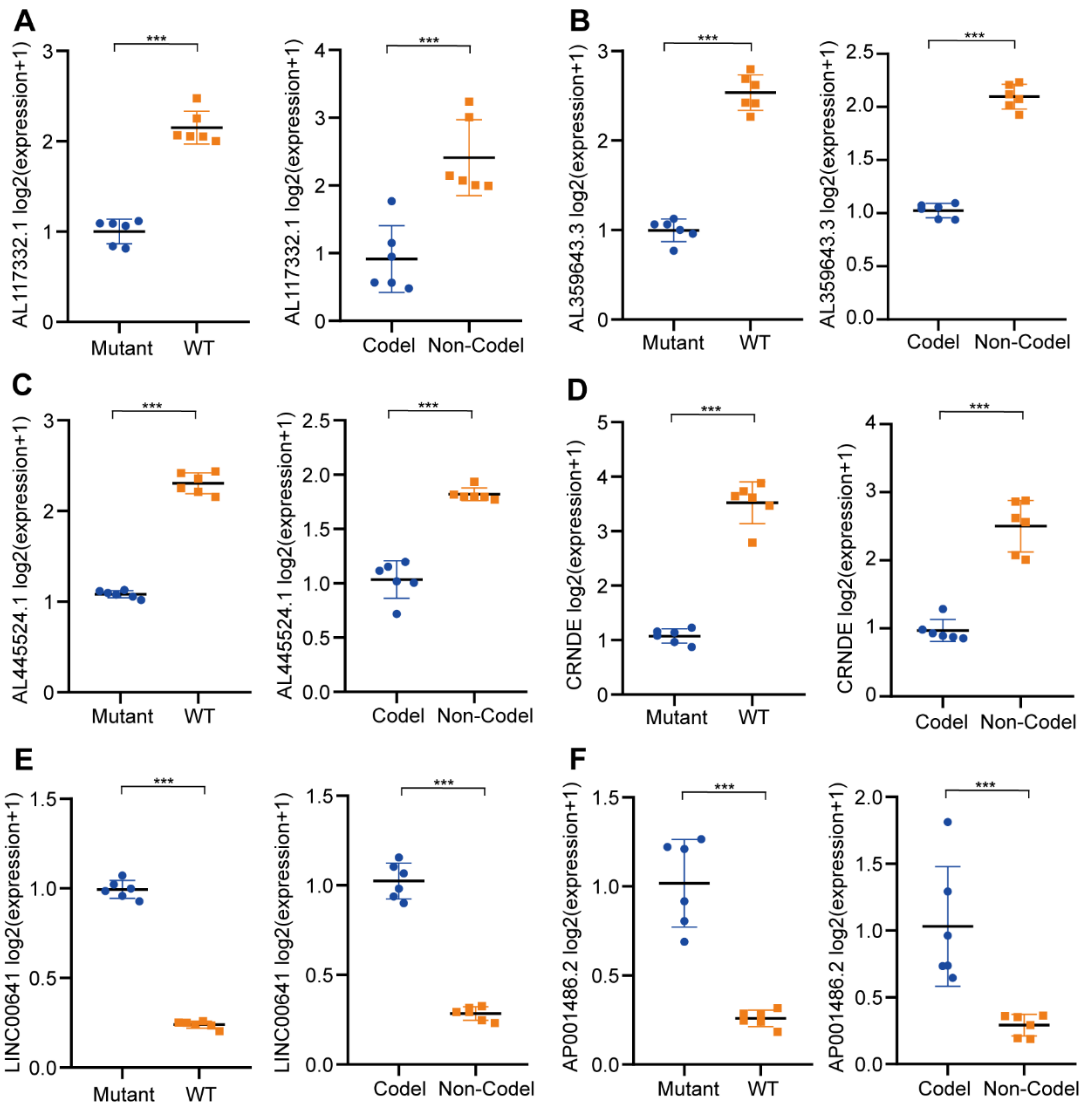

Supplementary Figure S14. Validation of the expression levels of m6A-associated lncRNAs in different glioma subgroups (IDH mutation status and 1p/19q codeletion status) by qRT-PCR. (**A-D**) The expression levels of AL1177332.1 (**A**), AL359643.3 (**B**), AL445524.1 (**C**), CRNDE (**D**) were much higher in the IDH-WT and 1p/19q-non-codel subgroups when compared to IDH-mutant and 1p/19q-codel subgroups. **(E-F**) The expression levels of LINC00641 (**E**) and AP001486.2 (**F**) in the IDH-mutant and 1p/19q-codel subgroups were much higher than in the IDH-WT and 1p/19q-non-codel subgroups.

Supplementary Table S1. Clinical features of glioma patients from TCGA

**Clinical features**		**Total (629)**	**%**
Age	Age <=46 (median age*)	316	50.24%
	Age >46 (median age)	313	49.76%
Gender	Female	270	42.93%
	Male	359	57.07%
Grade	LGG	477	75.83%
	GBM	152	24.17%
1p/19q	Non-codel	468	74.40%
	Codel	156	24.80%
	Unknow	5	0.80%
IDH	Mutant	400	63.59%
	WT	220	34.98%
	Unknow	9	1.43%
MGMT	Unmethylated	149	23.69%
	Methylated	448	71.22%
	Unknow	32	5.09%

*The median age was calculated from the clinical data of 629 glioma samples in TCGA datasets

Supplementary Table S2. Clinical features of glioma patients from CGGA

**Clinical features**		**Total (655)**	**%**
Age	Age <=46 (median age*)	411	62.75%
	Age >46 (median age)	244	37.25%
Gender	Female	282	43.05%
	Male	373	56.95%
Grade	LGG	419	63.97%
	GBM	236	36.03%
1p/19q	Non-codel	452	69.01%
	Codel	137	20.92%
	Unknow	66	10.07%
IDH	Mutant	332	50.69%
	WT	275	41.98%
	Unknow	48	7.33%
MGMT	Unmethylated	216	32.98%
	Methylated	304	46.41%
	Unknow	135	20.61%

*The median age was calculated from the clinical data of 655 glioma samples in CGGA datasets

Supplementary Table S3. 23 m6A-associated genes.

**Types**	**m6A-associated genes**
writers	METTL3, METTL14, METTL16, RBM15, RBM15B, VIRMA, WTAP, ZC3H13
readers	FMR1, HNRNPC, HNRNPA2B1, IGFBP1, IGFBP2, IGFBP3, LRPPRC, RBMX<br>YTHDC1, YTHDC2, YTHDF1, YTHDF2, YTHDF3
erasers	FTO, ALKBH5

Supplementary Table S4. 47 immune-checkpoint genes.

**Types**	**Gene names**
immune-checkpoint genes	IDO1、LAG3、CTLA4、TNFRSF9、ICOS、CD80、PDCD1LG2、TIGIT、CD70、TNFSF9、ICOSLG、KIR3DL1、CD86、PDCD1、LAIR1、TNFRSF8、TNFSF15、TNFRSF14、IDO2、CD276、CD40、TNFRSF4、TNFSF14、HHLA2、CD244、CD274、HAVCR2、CD27、BTLA、LGALS9、TMIGD2、CD28、CD48、TNFRSF25、CD40LG、ADORA2A、VTCN1、CD160、CD44、TNFSF18、TNFRSF18、BTNL2、C10orf54、CD200R1、TNFSF4、CD200 and NRP1

Supplementary Table S5. The primer sequences of genes.

Genes	Primer sequences
AL117332.1	Forward: 5′-CTTCTCCACCTGGGTGTCAC-3′,<br>Reverse: 5′- CAGGGTGCAGTGGAGTAGTG-3′;
AL359643.3	Forward: 5′-GAGTTTTCCTGAGGCCTCCC-3′,<br>Reverse: 5′- AGTCAATTCTCGCACTGCCA-3′;
AL445524.1	Forward: 5′-ACAACTACTTTCTGGCCGGG-3′,<br>Reverse: 5′-GGACCTTTGCCTGGCATTTG-3′;
CRNDE	Forward: 5′-CTCTAGTCGTGTCCCCTCGT-3′,<br>Reverse: 5′- TTCTGCGTGACAACTGAGGA-3′;
LINC00641	Forward: 5′-GGCTTCCCTGAGAGCAGATC-3′,<br>Reverse: 5′- AGCAAGGATGTGGAGCAACA-3′;
AP001486.2	Forward: 5′-ATGCATATGGGGAGGGGGAT-3′,<br>Reverse:5′-TATCCTGACCCATGCCTCCA -3′;
GAPDH	Forward:5′-GGTGTGAACCATGAGAAGTATGA-3′,<br>Reverse: 5′-GAGTCCTTCCACGATACCAAAG-3′;
